# Ivermectin Inhibits Stress Granule Clearance by Blocking the De Novo Synthesis of Hsp70 in Neuroblastoma Cells

**DOI:** 10.3390/cells15171623

**Published:** 2026-09-07

**Authors:** Siwei Chu, Elizabeth P. Anim, Reyhaneh Salehi-Tabar, John H. White, Ursula Stochaj

**Affiliations:** 1 Department of Physiology, McGill University, Montreal, QC H3G 1Y6, Canada; siwei.chu@mail.mcgill.ca (S.C.); elizabeth.anim@mail.mcgill.ca (E.P.A.); reyhaneh.salehitabar@mail.mcgill.ca (R.S.-T.); john.white@mcgill.ca (J.H.W.); 2 Quantitative Life Sciences Program, McGill University, Montreal, QC H3A 1E3, Canada

**Keywords:** ivermectin, stress response, stress granule, stress recovery, molecular chaperones, human cancer cells, Bioorthogonal Non-Canonical Amino Acid Tagging

## Abstract

**Highlights:**

**What are the main findings?**

- This study provides new mechanistic insights into the action of the anti-parasitic drug ivermectin in cancer cells.
- Ivermectin controls stress responses in a cell context-specific fashion.
- Ivermectin impairs the recovery from stress in human neuroblastoma cells.

**What are the implications of the main findings?**

- This work is important for the future use of ivermectin and its derivatives in basic research and as a lead compound for clinical applications.

**Abstract:**

Cytoplasmic stress granules (SGs) form in response to diverse insults; they are dismantled when the stress subsides. SG clearance is facilitated by molecular chaperones, nuclear transport factors, and other components. The anti-parasitic drug ivermectin inhibits nuclear trafficking and has potential anti-cancer activities. However, the molecular pathways that promote ivermectin’s therapeutic actions are poorly understood. Our study defined the effects of ivermectin on stress recovery in human neuroblastoma and cervical carcinoma cells. We demonstrate that ivermectin interferes with SG disassembly in neuroblastoma cells. The delay of SG dissolution is accompanied by significant changes in the proteostasis network. Notably, ivermectin diminishes de novo protein synthesis in unstressed and stressed cells. During recovery, ivermectin reduces the abundance of hsp70 in neuroblastoma, but not in cervical carcinoma cells. Surprisingly, ivermectin has no effect on Hsf1 abundance and localization. Moreover, ivermectin does not diminish the levels of transcripts encoding hsp70. Bioorthogonal Non-Canonical Amino Acid Tagging revealed that ivermectin markedly reduces the stress-induced de novo synthesis of hsp70 in neuroblastoma cells. Taken together, ivermectin can derail stress responses by a unique mechanism that alters the translation of hsp70 mRNA and is determined by the cellular context. This information is directly relevant to ivermectin-based anti-cancer therapies.

## 1. Introduction

Ivermectin is currently used in the clinic to treat parasitic infections in humans and animals [1,2,3]. Due to its health benefits, the WHO lists ivermectin as an essential medicine [4]. The drug and its derivatives have possible applications as anti-cancer and anti-viral agents [2,5,6,7]. The treatment potential of ivermectin relies, at least in part, on its ability to inhibit nuclear transport [8,9,10,11,12]. Specifically, ivermectin impairs the nuclear import of cargo proteins that are recognized by importin-α/importin-β1 [12]. Such cargoes carry a nuclear localization sequence (NLS) and include a wide variety of proteins that control the growth, proliferation, and survival of tumor cells.

Apart from their essential role in nucleocytoplasmic trafficking, nuclear transport factors are key players in the cellular response to endogenous and environmental stress. For example, importin-α and importin-β1 localize to cytoplasmic stress granules (SGs) when cells experience deleterious conditions [13]. Moreover, nuclear transport factors, such as importin-β1 and transportin-1 (*TNPO1*), facilitate the disassembly of persistent protein aggregates that can arise from SGs [14]. Additional links connect nuclear protein transport activities to granulostasis. For example, SG formation can be associated with the inhibition of nuclear import [15], but this may not universally apply to all cellular contexts [16]. On the other hand, failure to import specific RNA-binding proteins into the nucleus can facilitate the assembly of RNA granules in the cytoplasm [17]. Notably, some nuclear import factors are also critical for the localization of specific proteins to SGs [18].

SGs are non-membranous compartments that assemble in the cytoplasm of eukaryotic cells upon exposure to different insults, including oxidative stress and chemotherapeutic agents [13,19]. Following stress, the arrest of translation initiation promotes the transient formation of SGs. These biocondensates are enriched for RNA and RNA-binding proteins. For most stress conditions, SG-associated functions promote cell survival [13]. Notably, SGs are also implicated in the pathologies of cancer, neurodegeneration, and infectious and inflammatory diseases [13,19].

The homeostasis of RNA-protein granules, known as granulostasis, is regulated by multiple factors [20]. Molecular chaperones, nuclear transport receptors, and components of the autophagy apparatus are critical elements of the granulostasis network [21]. These factors regulate SG clearance when the stress subsides [22]. Molecular chaperones, especially members of the Hsp70 family, are key players in carcinogenesis, cancer cell progression, metastasis, and the development of chemoresistance [23]. Due to the “addiction” of cancer cells to chaperone function, heat shock proteins have become prominent targets for oncotherapy.

The potential of ivermectin for chemotherapeutic and other health applications prompted us to examine its effects on stress recovery in human neuroblastoma and cervical carcinoma cells. We selected these cells because they have been widely used to examine stress responses and proteostasis. Moreover, SH-SY5Y neuroblastoma cells are established model systems to study different aspects of neurodegeneration [24,25]. They have been useful in uncovering the roles of RNA granules in neurodegenerative diseases [26].

Our study revealed that ivermectin alters granulostasis in cancer cells. Importantly, the responses to ivermectin differ significantly in distinct cancer cell lines. In particular, ivermectin-treated neuroblastoma cells failed to increase the abundance of the molecular chaperone Hsp70 during stress recovery. We showed that ivermectin inhibits the translation of Hsp70 mRNA in neuroblastoma, but not in cervical carcinoma cells. This knowledge is important for the future use of ivermectin as a lead molecule in anti-cancer drug development.

## 2. Materials and Methods

### 2.1. Reagents

Ivermectin (MK-933, IVM) was purchased from Selleckchem (Houston, TX, USA). Primary and secondary antibodies were obtained from the suppliers listed in Table 1. L-Azidohomoalanine (BP-23383) and DBCO-PEG4-biotin (BP-22295) were from BroadPharm (San Diego, CA, USA). Streptavidin magnetic beads were obtained from GenScript (L00936).

### 2.2. Cell Culture and Exposure to Oxidative Stress

SH-SY5Y (human neuroblastoma) and HeLa (human cervical carcinoma) cells were initially obtained from ATCC. Cells were cultured in Dulbecco’s modified Eagle’s medium (DMEM) supplemented with 8% bovine calf serum (BCS, Fisher Scientific, Ottawa, ON, Canada) and 1% penicillin/streptomycin [27]. Cells were free from mycoplasma contamination; they were tested regularly with a commercial kit (Applied Biological Materials, abm, Richmond, BC, Canada). SG formation was induced by incubation with 0.5 mM sodium arsenite (referred to as arsenite) for 30 min at 37 °C; distilled water served as the vehicle control. The recovery was performed in standard growth medium (DMEM, 8% BCS, 1% penicillin/streptomycin). For Bioorthogonal Non-Canonical Amino Acid Tagging (BONCAT) experiments, cells were cultured as described by us [28]. We have published a detailed BONCAT protocol describing each step of the procedure [29].

### 2.3. Cell Viability Assay

Cell viability was measured with a resazurin-based assay [30].

### 2.4. Transfection and Evaluation of Nuclear Protein Import

We described earlier the use of fluorescent reporter proteins for nuclear import [31]. Here, SH-SY5Y and HeLa cells were transfected with plasmids encoding mCitrine fused to the SV40 nuclear localization signal (NLS) or coding for the mCitrine tag. The K2 transfection kit was used as recommended by the manufacturer (Biontex, München, Germany). Following transfection, cells were fixed with 3.7% formaldehyde in PBS, washed in PBS, and DNA was visualized with 4′,6-diamidino-2-phenylindole (DAPI). Fluorescence intensities in the nucleus and cytoplasm were quantified with ImageJ (2.16.0/1.54m) [27]. In brief, after background correction, pixel intensities/area were quantified for the region of interest.

### 2.5. Immunofluorescence

Cells were grown on poly-lysine-coated coverslips and incubated with vehicle, arsenite, ivermectin or ivermectin plus arsenite (see Results section). Following treatment, cells were fixed and processed for immunostaining. We have published a detailed protocol earlier [32]. The measurement of pixel intensities/area was performed as described in Section 2.4. The concentrations of primary and secondary antibodies are listed in Table 1.

### 2.6. Crude Cell Extracts, SDS-Polyacrylamide Gel Electrophoresis, and Western Blotting

The preparation of crude cell extracts, separation of samples by denaturing gel electrophoresis, Western blotting, and acquisition of signals for enhanced chemiluminescence (ECL) followed our published procedures [30]. ECL images may have been obtained from the same blot for more than one antibody. For these antigens, the actin Western blots will be identical. Please note that the calculation of ECL signals corrects for possible variable loading of the gel. For example, many of our ECL data were normalized to actin. Alternatively, when appropriate, the bar graphs depict the ratio of a phosphorylated protein/total protein.

### 2.7. RNA Extraction, Reverse Transcription, and qPCR

SH-SY5Y and HeLa cells were seeded in 6-well plates with a seeding density of approximately 70%. After treatment, cells were rinsed with PBS, and total RNA was extracted with a total RNA mini kit (ALPHAGEN, Beloeil, QC, Canada) as per the manufacturer’s instruction. Total RNA was quantified with a NanoDrop 2000c (Thermo Fisher Scientific, Saint-Laurent, QC, Canada). RNA was converted to cDNA using the AdvanTech 5× master mix. Each qPCR reaction was performed with 10 ng cDNA in BlasTaq 2× qPCR MasterMix (abm, Richmond, BC, Canada). qPCR reactions were conducted with a Stratagene MX3005P (Agilent, Mississauga, ON, Canada) machine. Gene expression was normalized to the expression level of *ACTB*. Fold changes were calculated as 2^−ΔΔCt^. The primers used for qPCR are listed in Table 2.

### 2.8. Statistical Evaluation

A minimum of *n* = 3 independent experiments were carried out for viability tests and Western blotting. To assess more than two experimental groups, the statistical evaluation was performed with One-way Analysis of Variance (ANOVA) combined with Bonferroni post hoc analysis. Differences were considered significant for *p* < 0.05. Differences between controls and treated cells are marked with *, *p* < 0.05; **, *p* < 0.01; ***, *p* < 0.001. Significant differences between other pairs of the dataset are labeled as follows: #, *p* < 0.05; ##, *p* < 0.01; ###, *p* < 0.001. Student’s *t*-test was applied to compare two experimental groups. For instance significant differences between SH-SY5Y and HeLa cells are shown as: $, *p* < 0.05; $$, *p* < 0.01; $$$, *p* < 0.001. Further details on the number of independent experiments and statistical methods are included in the figure legends.

## 3. Results

### 3.1. Ivermectin Interferes with Classical Nuclear Import in SH-SY5Y and HeLa Cells

Ivermectin impairs the interaction between importin-α and importin-β1, which compromises classical nuclear import and could be lethal [2]. Based on our toxicity evaluation for SH-SY5Y neuroblastoma and HeLa cervical carcinoma cells (Figure S1a), we selected 50 µM ivermectin for subsequent experiments. This concentration elicited negligible to low adverse effects in either cell line. Moreover, a 3 h treatment with 50 µM ivermectin did not trigger cell death, either alone or when combined with the oxidative stressor arsenite (Figure S1b). To determine the impact of ivermectin on nuclear trafficking, cells were transfected with plasmids coding for either mCitrine fused to the classical SV40-NLS (NLS-mCitrine) or mCitrine alone (Figure S2). Nuclear import inhibition by ivermectin was apparent in both cell lines for NLS-mCitrine. By contrast, ivermectin did not alter the distribution of mCitrine (Figure S2).

### 3.2. Ivermectin Delays the Clearance of Stress Granules in SH-SY5Y Neuroblastoma Cells

Importin-α1 is required for the proper assembly of arsenite-induced SGs [33]. Furthermore, importin-α and importin-β1 are present in oxidant-induced SGs [34], and ivermectin impairs nuclear transport (Figure S2). As nuclear transport factors control granulostasis, it was conceivable that ivermectin alters SG properties. Since ivermectin does not prevent SG formation (Chu et al., unpublished), we focused on the removal of SGs during the recovery from stress. Oxidative stress was induced with arsenite, a widely used agent to generate SGs.

**Figure 1:**
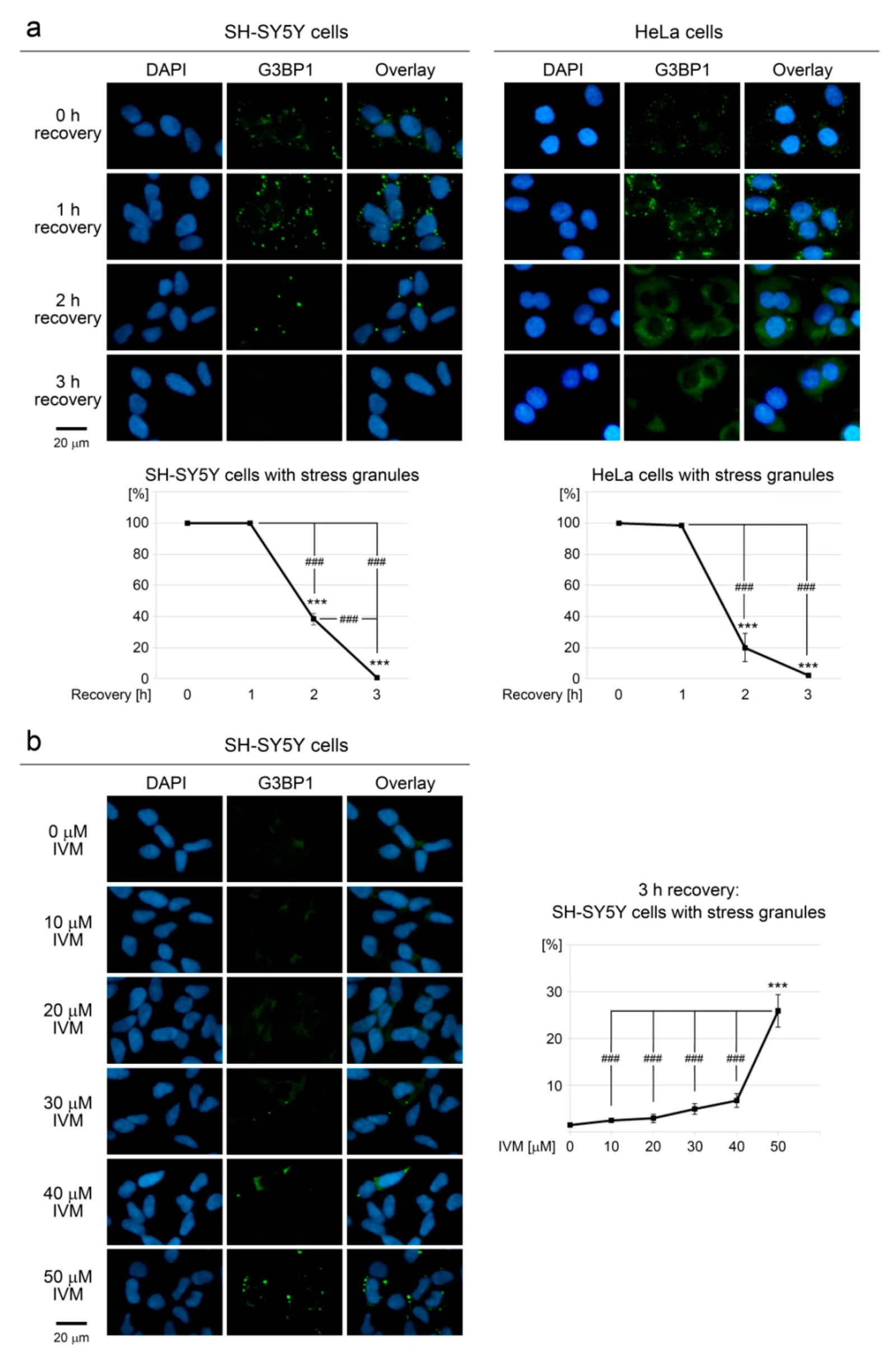
(**a**) **Stress granule clearance is controlled by the cell context.** SH-SY5Y and HeLa cells were exposed to arsenite stress. After removal of arsenite, the percentage of cells containing SGs was determined at the recovery times indicated. The marker protein G3BP1 identified SGs. The graphs show results (average ± SEM) for *n* = 3 independent experiments. At least 100 cells were scored per experiment for each condition. One-way ANOVA combined with Bonferroni correction detected significant differences. The 0 h recovery point served as reference; ***, *p* < 0.001. Pairwise comparisons identified significant differences between two conditions; ###, *p* < 0.001. (**b**) **Ivermectin delays stress granule removal in SH-SY5Y cells.** Different ivermectin concentrations were evaluated for their effect on SG dissolution. G3BP1 served as SG marker. Image quantification and statistical analyses were performed as described for part (**a**). Size bars in part (**a**,**b**) are 20 µm.

We evaluated SG removal (Figure 1) employing the SG-nucleating factor Ras-GTPase-Activating Protein SH3-Domain-Binding Protein 1 (G3BP1) as a marker protein (Figure 1a). After arsenite withdrawal, SGs fused and became more prominent during the first hour of recovery. Extending the recovery period to 2 h or 3 h significantly reduced the percentage of cells with SGs. The kinetics of SG dissolution was dependent on the cellular context. At 2 h, SG clearance was more effective in HeLa cells. Following a 3 h recovery period, SGs were efficiently dispersed in neuroblastoma and cervical carcinoma cells (Figure 1a).

To examine its effects on SG clearance, ivermectin was added at the beginning of the recovery period. After 3 h, the drug significantly delayed the dismantling of SGs in SH-SY5Y (Figure 1b), but not in HeLa cells (not shown). Treatment with 50 µM ivermectin alone for 3 h did not elicit SG assembly in either cell line.

**Figure 2:**
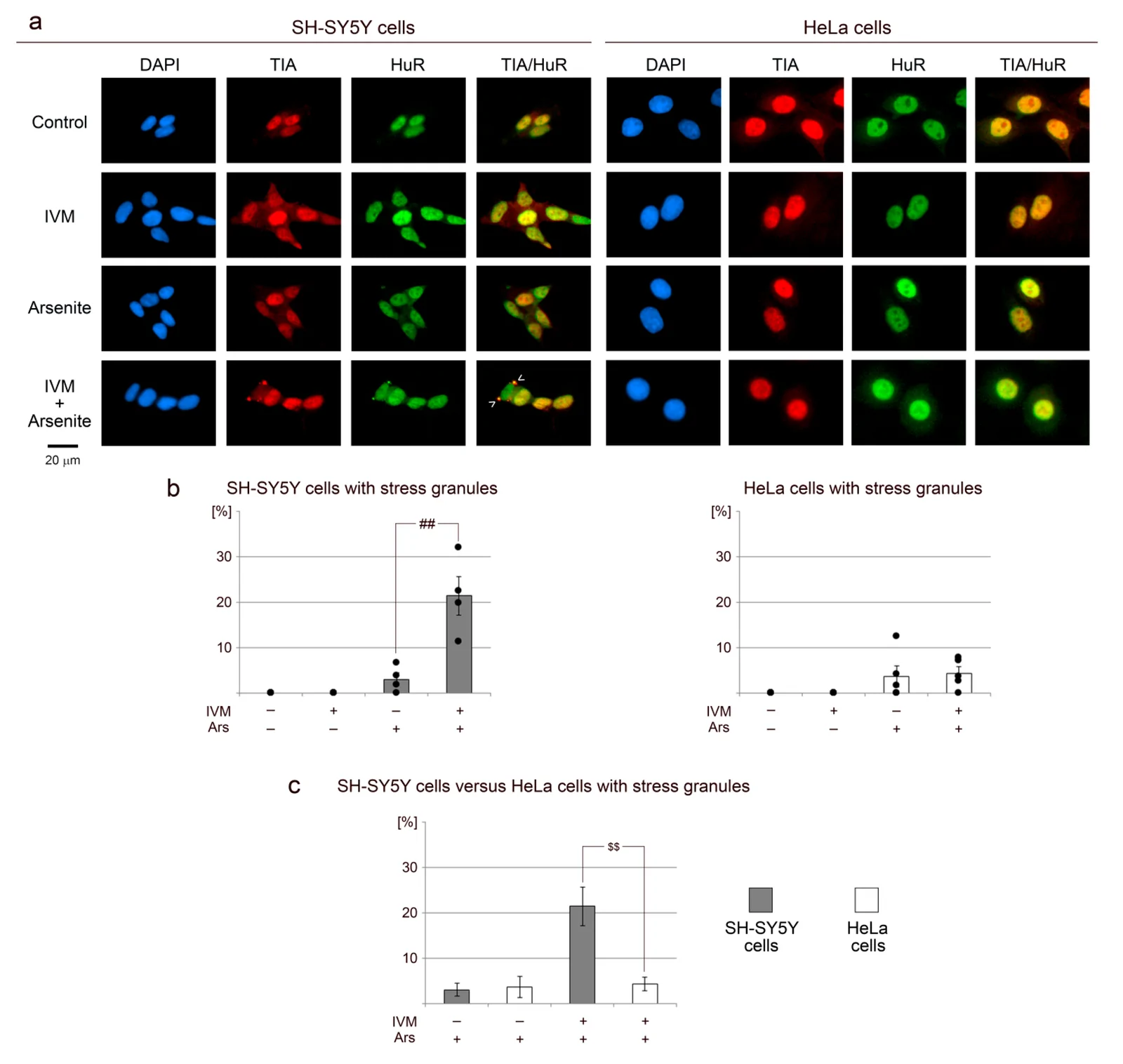
**Ivermectin delays stress granule clearance in neuroblastoma cells.** SH-SY5Y and HeLa cells were incubated with vehicle or arsenite (Ars), followed by a 3 h recovery period without or with ivermectin (IVM). (**a**) The presence of SGs was monitored with two different SG markers, TIA and HuR. The position of two SGs in neuroblastoma cells is marked by white arrowheads. Size bar is 20 µm. (**b**) The percentage of cells containing SGs was determined at the end of the 3 h recovery period. The scoring was performed with TIA as a granule marker. The graphs show results (average ± SEM) for *n* ≥ 4 independent experiments. At least 100 cells were assessed per experiment for each condition. Student’s *t*-test identified significant differences between arsenite-stressed cells that recovered in the absence or presence of ivermectin; ##, *p* < 0.01. (**c**) The presence of SGs in arsenite-stressed SH-SY5Y and HeLa cells was compared after a 3 h recovery without or with ivermectin, as described for part (**b**). Student’s *t*-test was used to detect significant differences between SH-SY5Y and HeLa cells $$, *p* < 0.01.

The effects of ivermectin on SG disassembly were independently examined with a different set of SG proteins, TIA and HuR (Figure 2a). Using TIA as a granule marker, we quantified the number of cells that contained SGs at the end of the recovery period (Figure 2b,c). Consistent with the results depicted in Figure 1, ivermectin significantly reduced SG disassembly in neuroblastoma cells (Figure 2b). By contrast, ivermectin did not delay granule dissolution in cervical carcinoma cells. Further evaluation revealed that SG clearance was significantly different in SH-SY5Y and HeLa cells when the stress recovery took place in the presence of ivermectin (Figure 2c). Together, the results in Figure 2 demonstrate that the effects of ivermectin on granule dynamics were consistently observed with different SG marker proteins.

Collectively, our experiments show that ivermectin significantly impacts stress recovery in neuroblastoma cells. Remarkably, cancer cells of diverse origin display marked differences in stress recovery. We hypothesized that ivermectin affects the granulostasis network of SH-SY5Y cells, which delays SG removal. The following experiments test this idea.

### 3.3. Ivermectin Modulates Stress Signaling

**Figure 3:**
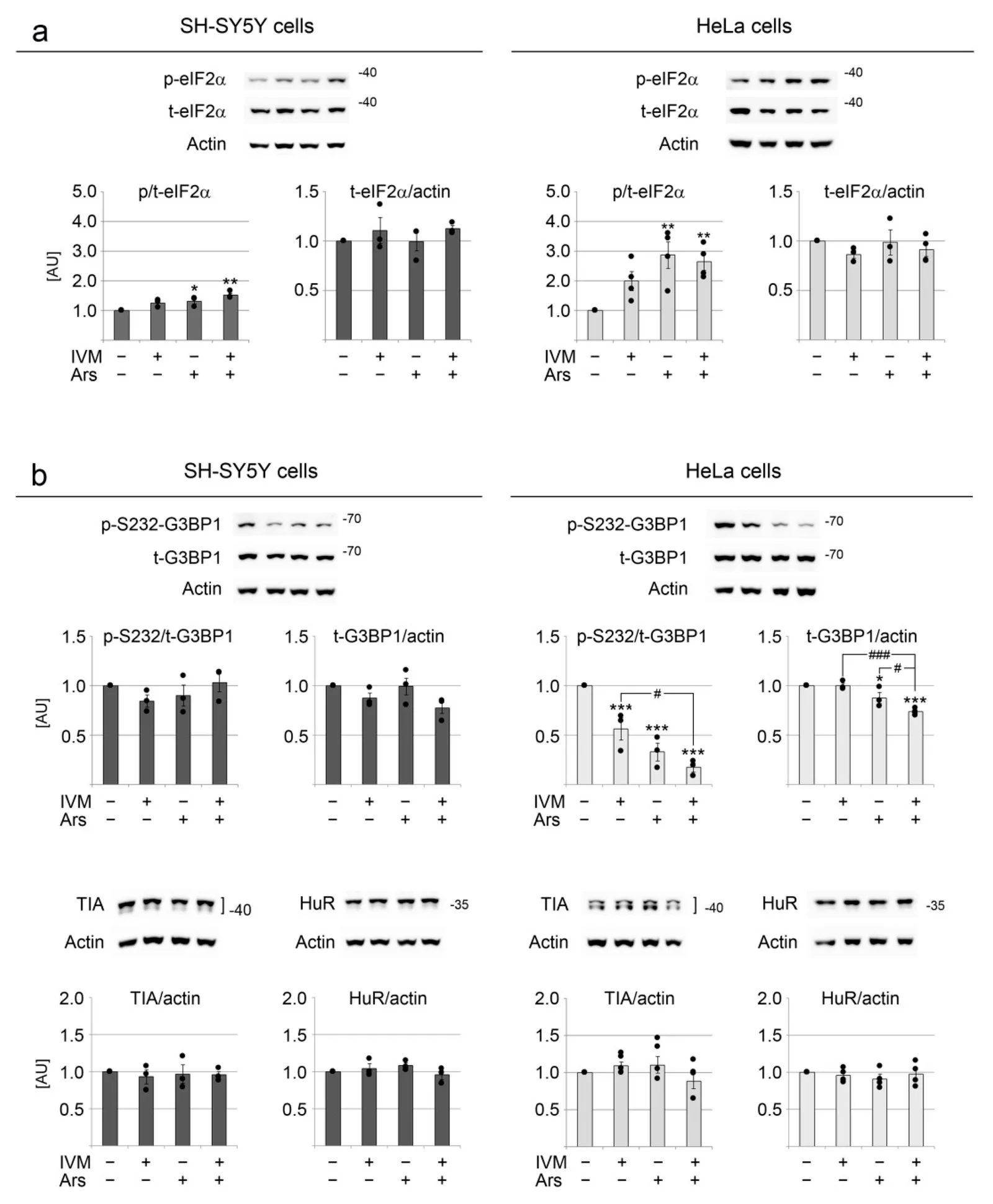
(**a**) **Ivermectin alters stress signaling in a cell-context-dependent fashion.** SH-SY5Y and HeLa cells were incubated with vehicle or arsenite (Ars). Vehicle (-IVM) or ivermectin (+IVM) was present throughout the subsequent 3 h stress recovery period. The preparation of cell extracts and Western blotting are described in the Materials and Methods section. (**b**) **Ivermectin controls the phosphorylation and abundance of G3BP1 in HeLa cells.** We assessed G3BP1 phosphorylation on S232 (p-S232), and the levels of total G3BP1 (t-G3BP1), TIA, and HuR. (**a**,**b**) Results for Western blotting were normalized to the vehicle control (-IVM, -Ars). Graphs depict averages ± SEM in arbitrary units, AU. Statistically significant differences were determined by One-way ANOVA plus Bonferroni post hoc correction. Comparisons were made pairwise to the control (-IVM, -Ars); *, *p* < 0.05; **, *p* < 0.01, ***, *p* < 0.001 or between treatment groups #, *p* < 0.05, ###, *p* < 0.001. Note that the same blot was used to monitor the abundance of HuR and PKR (Figure S3) in SH-SY5Y cells. The actin panels are therefore identical in both figures.

Multiple branches of the granulostasis network are involved in stress recovery. This prompted us to examine stress signaling, SG proteins, nuclear transport factors, autophagy, and molecular chaperones (Figure 3a,b and Figure S3 and below). We addressed cell context specific differences of the granulostasis network by comparing directly the protein levels in SH-SY5Y and HeLa cells (Figures S3 and S4, Supplemental Figures below).

The phosphorylation of eIF2α on S51 (p-S51-eIF2α) is mandatory for the assembly of canonical SGs [13]. Ivermectin and arsenite, either alone or in combination, elevated the ratio of p-S51-eIF2α/total eIF2α (Figure 3a). The increase in eIF2α phosphorylation was more pronounced in HeLa than in SH-SY5Y cells. We did not detect significant changes in the abundance of the catalytic subunit of protein phosphatase 1 (PP1), which dephosphorylates p-S51-eIF2α (Figure S3). GADD34/PPP1R15A and CReP/PPP1R15B are regulatory subunits of PP1 and modulate the dephosphorylation of p-S51-eIF2α [35]. Ivermectin had no significant effect on the levels of GADD34 (Figure S3). Commercial antibodies against CReP were unreliable; therefore, CReP was not assessed.

Protein kinase R (PKR) phosphorylates eIF2α on S51 and is activated by viral infections or oxidative stress, including arsenite [36]. Moreover, G3BP1 and PKR collaborate to activate antiviral innate immune responses [37]. As ivermectin has potential antiviral activities, we examined PKR in SH-SY5Y and HeLa cells. Ivermectin and arsenite elicited more pronounced PKR phosphorylation (i.e., activation) in HeLa cells (Figure S3).

Phosphoprotein phosphatase 2 A (PP2A) is a downstream target of PKR and responsive to oxidative stress [38]. Ivermectin and arsenite, either alone or in combination, elevated the abundance of PP2A in SH-SY5Y, but not in HeLa cells (Figure S3). Taken together, ivermectin does not abolish the phosphorylation of eIF2α or PKR, which are crucial signaling events in the formation of canonical SGs.

To uncover cell context-dependent differences, we compared the abundance of stress signaling factors in SH-SY5Y and HeLa cells. While PP1 and PP2A levels were significantly higher in neuroblastoma cells, GADD34 was significantly lower (Figure S4). Nevertheless, the stress-induced phosphorylation of eIF2α on S51 and total eIF2α were comparable in both cell lines.

In summary, eIF2α or PKR phosphorylation did not correlate with the presence of SGs in SH-SY5Yand HeLa cells. This suggests ivermectin reduces SG clearance in neuroblastoma cells by targeting other factors.

### 3.4. Ivermectin Regulates G3BP1 Phosphorylation in HeLa Cells, but Has Minor Effects on the Abundance of SG Proteins

Changes in the levels of SG proteins correlate with altered granulostasis [32]. G3BP1 and TIA are critical for SG formation, as they nucleate granules [39]. Ivermectin alone or combined with arsenite slightly diminished the abundance of G3BP1 in SH-SY5Y cells (Figure 3a). By contrast, HeLa cells displayed a small but significant loss of G3BP1 for arsenite/ivermectin treatment. Post-translational modifications of SG-nucleating proteins, especially G3BP1, control granule assembly. The phosphorylation of S149 and S232, residues adjacent to an internally disordered region, can modulate the liquid–liquid phase separation of G3BP1, which takes place during SG formation [40]. Data on the role of S149 phosphorylation for SG assembly are conflicting [40,41]. As commercial antibodies against phospho-S149-G3BP1 have low specificity (unpublished), we focused on residue S232. Ivermectin and arsenite significantly reduced S232 phosphorylation in HeLa cells, which increases G3BP1’s potential to generate biocondensates [40]. The combination arsenite/ivermectin led to the most profound loss of S232 phosphorylation. Arsenite and arsenite/ivermectin also diminished the abundance of total G3BP1 in HeLa cells. No significant changes were observed for TIA or HuR in either cell line (Figure 3b).

Interestingly, the relative abundance of SG-nucleating proteins G3BP1 and TIA differed significantly in neuroblastoma and cervical carcinoma cells (Figure S5). SH-SY5Y cells had lower levels of G3BP1, but higher amounts of TIA. Collectively, our data support the model that the loss of G3BP1 phosphorylation on S232 does not drive the persistence of SGs in neuroblastoma cells during stress recovery.

### 3.5. Ivermectin Has Minor Effects on Nuclear Transport Factors and the Autophagy-Related Protein LC3β

Nuclear transport factors, such as transportin-1 or importin-β1, contribute to the dismantling of protein aggregates [14]. Thus, ivermectin could impact SG dissolution by targeting nuclear transport components. During stress recovery, ivermectin affected the abundance of nuclear transport factors in neuroblastoma and cervical carcinoma cells (Figure S6). However, the changes were not significant. Side-by-side analyses of both cell lines revealed that HeLa cells contained higher levels of importin-α1 and transportin-1 (Figure S7). These differences may limit the accumulation of protein aggregates in HeLa cells.

The autophagy-lysosomal pathway can dismantle SGs, a process called granulophagy [22,42]. Granulophagy becomes more important when granules transition to a persistent state [22]. The conversion of LC3β-I to the membrane-bound LC3β-II form is a key step during autophagy. When recovering from arsenite stress, neuroblastoma cells elevated the LC3β-II/LC3β-I ratio (Figure S8a). Ivermectin diminished this increase only slightly. Interestingly, LC3β was far more abundant in SH-SY5Y cells than in HeLa cells (Figure S8b). (It should be noted that our study did not examine autophagic flux.)

In summary, the nuclear transport factors analyzed and LC3β were not strong candidates to mediate the ivermectin-induced block of SG clearance in neuroblastoma cells. This motivated us to focus on molecular chaperones and their co-factors, which are pillars of granulostasis.

### 3.6. Ivermectin Alters the Chaperone Network That Controls Granulostasis

**Figure 4:**
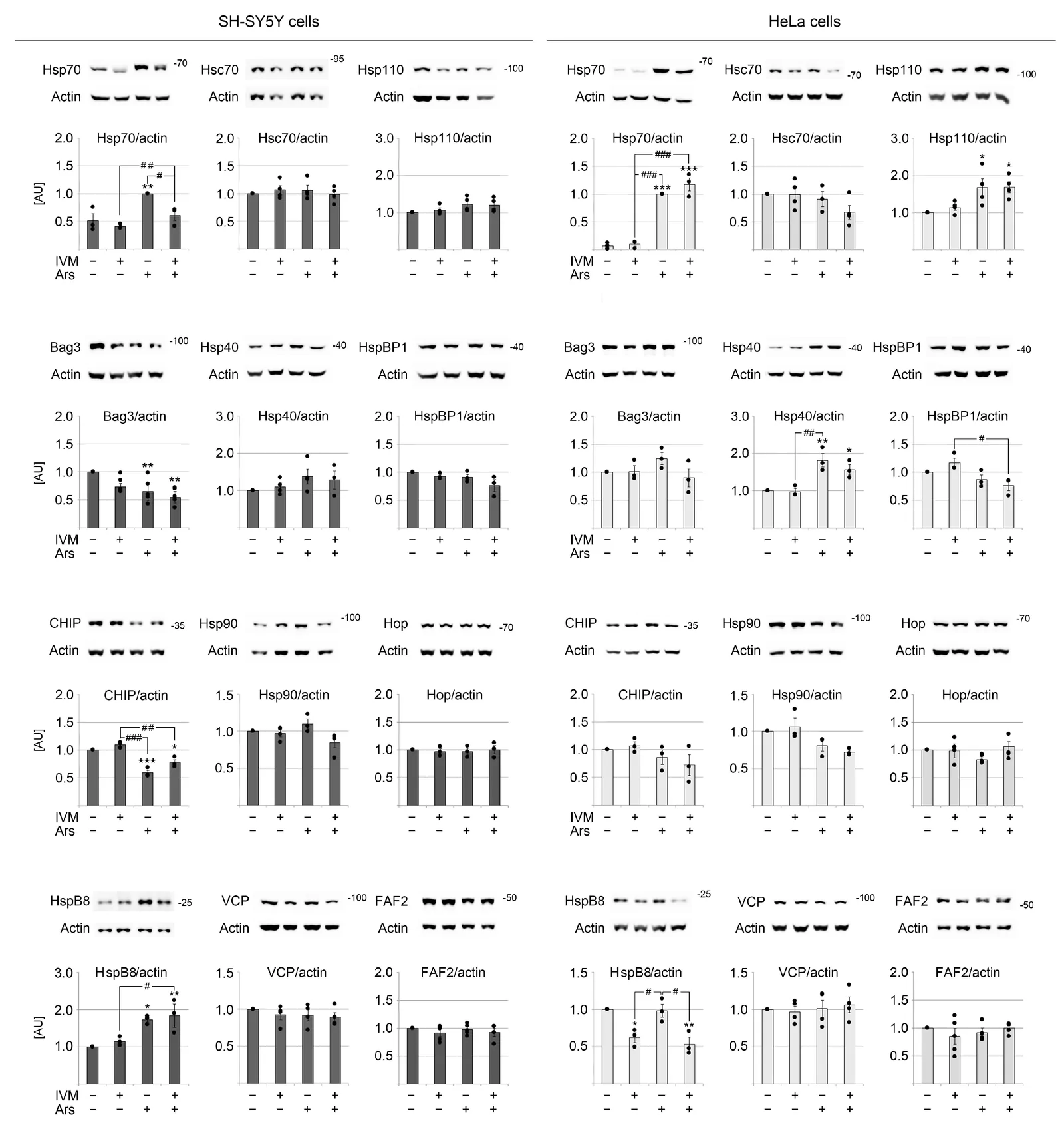
**Effect of ivermectin on the abundance of molecular chaperones and co-factors that control granulostasis.** Cells were treated as described for Figure 3a. The abundance of molecular chaperones was quantified for at least three (*n* ≥ 3) independent experiments. The molecular mass of marker proteins in kD is indicated at the right margins of blots. Results were normalized to the vehicle control (-IVM, -Ars). Graphs show averages ± SEM. Statistically significant differences were determined by One-way ANOVA plus Bonferroni correction. Comparisons were made pairwise to the control (-IVM, -Ars); *, *p* < 0.05; **, *p* < 0.01; ***, *p* < 0.001 or between treatment groups #, *p* < 0.05, ##, *p* < 0.01, ###, *p* < 0.001. AU, arbitrary units.

Hsp70 and its co-chaperones regulate granulostasis [20]. The combined action of Hsp70, Bag3, and HspB8 is especially important for granule disassembly [43]. VCP and its co-factors, such as FAF2, also control SG clearance, at least for some stress conditions [44]. Figure 4 evaluates a panel of granulostasis factors [44,45,46,47]. During stress recovery, Hsp70 levels increased in both SH-SY5Y and HeLa cells. Ivermectin significantly abolished this increase in neuroblastoma, but not in cervical carcinoma cells.

The co-chaperone Hsp110 stimulates the removal of protein aggregates [48,49], and Hsp40 family members are implicated in SG clearance [22]. However, ivermectin did not induce significant changes for Hsp110 and Hsp40 during stress recovery. Furthermore, the effects on CHIP, HspBP1, Hsp90, Hop, VCP, and FAF2 were only minor (Figure 4).

The abundance of HspB8 was increased by arsenite and arsenite/ivermectin in neuroblastoma cells, but diminished by ivermectin and arsenite/ivermectin in HeLa cells. Interestingly, SH-SY5Y cells contained much less HspB8 than HeLa cells (Figure S9). We further validated this result with a different antibody that was raised in another species (Figure S10).

Collectively, our results show that ivermectin alters the chaperone network during stress recovery. The severe drop of Hsp70 levels in SH-SY5Y cells is expected to impair the disposal of SGs [20,50]. Our data strengthen the model that cell context determines granulostasis network properties and thereby the cellular response to stress.

### 3.7. Ivermectin Regulates Hsf1 Phosphorylation and Increases Hsp70 Transcript Abundance

**Figure 5:**
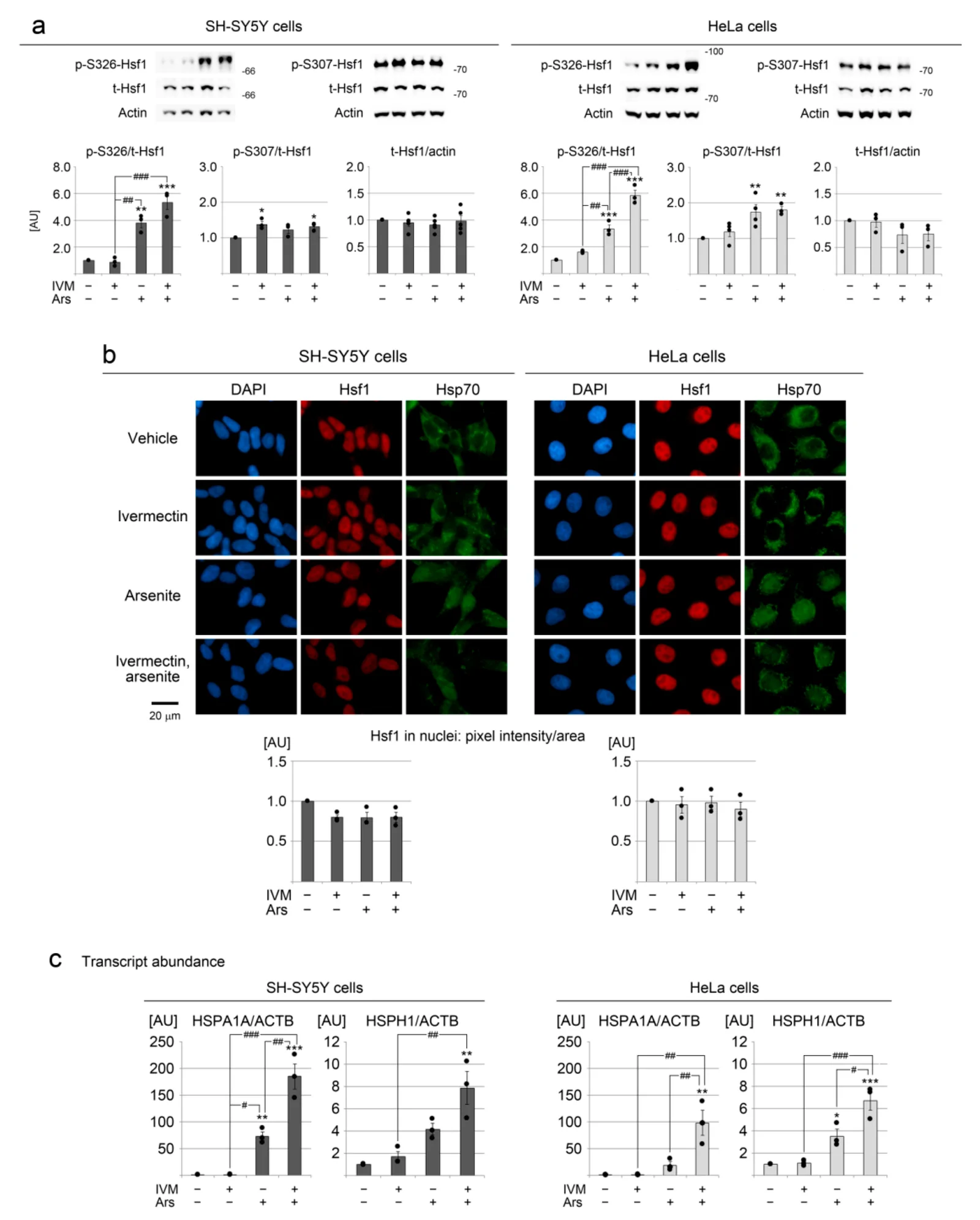
**Ivermectin modulates Hsf1 phosphorylation and increases the abundance of chaperone mRNA during stress recovery.** SH-SY5Y and HeLa cells were incubated with ivermectin (IVM) and arsenite (Ars), as shown in the figure. (**a**) Results for Western blotting represent a minimum of three independent experiments (*n* ≥ 3). The molecular mass of marker proteins in kD is shown at the right margins of blots. (**b**) Hsf1 was detected by immunolocalization, and the pixel intensity in nuclei was quantified for *n* = 3 independent experiments. Size bar is 20 µm. (**c**) The relative abundance of *HSPA1A* and *HSPH1* transcripts was quantified as detailed in the Materials and Methods section. Data represent *n* = 3 independent experiments. (**a**–**c**) All graphs depict the average of results ± SEM. Significant differences were identified by One-way ANOVA plus Bonferroni correction. Pairwise comparisons were made to the control (-IVM, -Ars); *, *p* < 0.05; **, *p* < 0.01; ***, *p* < 0.001; or between treatment groups #, *p* < 0.05, ##, *p* < 0.01; ###, *p* < 0.001. AU, arbitrary units.

The transcription factor Hsf1 upregulates the expression of *HSP70* genes in response to stress [51]. Figure 5 determines the impact of ivermectin and arsenite on Hsf1 phosphorylation, abundance, and localization. The transcriptional activity of Hsf1 depends on post-translational modifications. Stress promotes Hsf1 phosphorylation on S326 [52], which stimulates Hsf1-dependent transcription. By contrast, S307 phosphorylation reduces the transcriptional activity of Hsf1 [53,54].

Hsf1 phosphorylation on S326 was comparable in SH-SY5Y and HeLa cells (Figure 5a; p-S326-Hsf1). P-S326-Hsf1 significantly increased during stress recovery; this was further amplified by ivermectin. Arsenite and arsenite/ivermectin treatments elevated p-S307-Hsf1 abundance in HeLa, but not in SH-SY5Y cells. Total Hsf1 levels did not significantly change in either cell line. Thus, differences in the phosphorylation of key Hsf1 sites, S326 and S307, do not explain the ivermectin-induced loss of Hsp70 protein in recovering neuroblastoma cells. The dephosphorylation by PP2A is needed for Hsf1 to be fully transcriptionally active [55]. The catalytic PP2A subunit increased for arsenite and arsenite/ivermectin in neuroblastoma cells (Figure S3). This indicates that ivermectin does not limit Hsf1 activation during recovery.

SH-SY5Y and HeLa cells differ in Hsf1 phosphorylation, but not in the total amount of Hsf1 (Figure S11). Specifically, the stress-induced phosphorylation of both S326 and S307 was significantly higher in neuroblastoma than in HeLa cells.

The classical nuclear import apparatus mediates Hsf1 nuclear trafficking under nonstress conditions [56]. Cell fractionation data indicate that stress enhances the nuclear accumulation of Hsf1; immunolocalization studies contradict these results [57]. Since nuclear contents often leak into the cytoplasm during cell fractionation, we examined the subcellular distribution of Hsf1 by quantitative immunofluorescence (Figure 5b). This method clearly showed that Hsf1 was concentrated in nuclei under all conditions tested. None of the treatments caused significant changes in Hsf1 subcellular distribution in neuroblastoma or cervical carcinoma cells.

HspA1A is a canonical member of the Hsp70 family; its mRNA and protein levels are highly elevated upon stress [58,59]. To explore the ivermectin-dependent mechanisms that control Hsp70 protein abundance, *HSPA1A* transcripts were measured. For comparison, we also evaluated *HSPH1* transcripts, which encode Hsp110 (Figure 5c). The mRNAs for both proteins increased significantly during stress recovery, and ivermectin further amplified this change. Of note, the stress-dependent rise in transcript abundance was far more pronounced for *HSPA1A* than for *HSPH1.*

Taken together, ivermectin enhanced the activating phosphorylation of Hsf1 on S326 during stress recovery in SH-SY5Y and HeLa cells. This event was accompanied by an increase in *HSPA1A* mRNA in both models. We conclude that Hsf1 abundance, activation, localization, or transcriptional activity do not explain the striking effect of ivermectin on Hsp70 protein levels in recovering neuroblastoma cells.

### 3.8. Ivermectin Inhibits the De Novo Protein Synthesis of Hsp70 During Stress Recovery in SH-SY5Y, but Not in HeLa Cells

**Figure 6:**
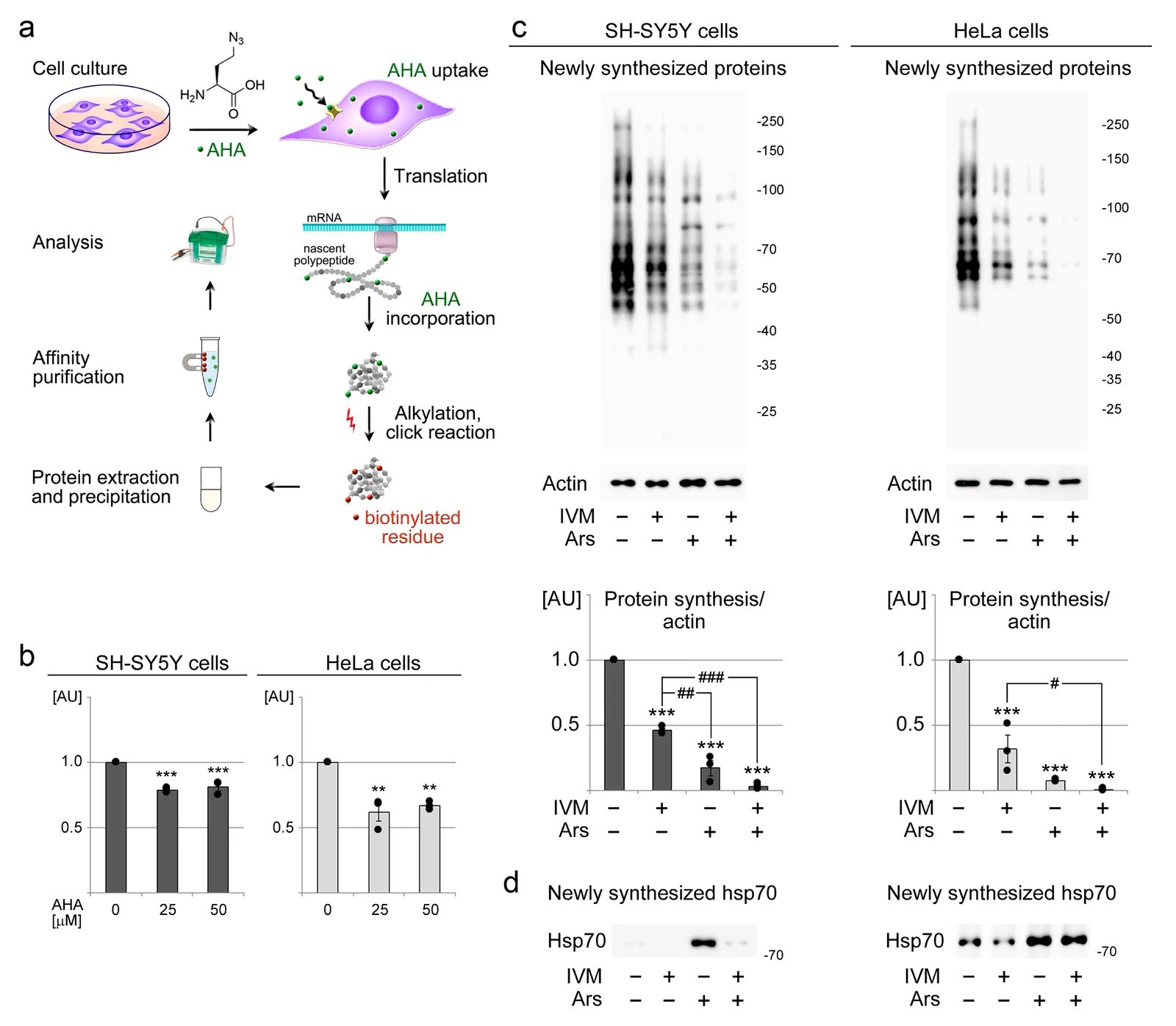
**Ivermectin inhibits the translation of Hsp70 mRNA in SH-SY5Y cells recovering from arsenite stress.** (**a**) Schematic representation of the BONCAT workflow. See Materials and Methods section for details. (**b**) Toxicity of L-azidohomoalanine in SH-SY5Y and HeLa cells. Cells were incubated with medium supplemented with L-methionine (0 µM AHA), 25 µM or 50 µM L-azidohomoalanine (AHA). The cell viability was measured after 24 h. Graphs represent the averages ± SEM for *n* = 3 independent experiments. Statistical evaluation was performed with One-way ANOVA plus Bonferroni correction. Comparisons were made pairwise to the control (0 µM AHA); **, *p* < 0.01; ***, *p* < 0.001. AU, arbitrary units. (**c**) Neuroblastoma and cervical carcinoma cells were treated with ivermectin (IVM) and arsenite (Ars), followed by BONCAT labeling during stress recovery. Biotinylated proteins were detected with Streptavidin-HRP, and ECL signals were quantified for *n* = 3 independent experiments. Signals were normalized to the total amount of actin present in each lane. One-Way ANOVA combined with Bonferroni correction identified significant differences between datasets. Comparisons were performed pairwise to the control (-IVM, -Ars); ***, *p* < 0.001; or between treatment groups #, *p* < 0.05, ##, *p* < 0.01; ###, *p* < 0.001. AU, arbitrary units. (**d**) The presence of newly synthesized Hsp70 was assessed by Western blotting of biotinylated proteins and probing with antibodies against Hsp70. The loading in part (**d**) was equivalent to the amounts of protein separated in part (**c**). The blots show representative results of *n* = 3 independent experiments.

Surprisingly, during stress recovery, the mRNA levels (Figure 5c) did not correlate with Hsp70 protein abundance in neuroblastoma cells (Figure 4). Therefore, we explored how ivermectin impacts mRNA translation. This was achieved with a Bioorthogonal Non-Canonical Amino Acid Tagging (BONCAT) approach we developed for mammalian cells [28,29]. Figure 6a provides a schematic overview of the method. Based on our toxicity assessment (Figure 6b), all subsequent experiments were conducted with 50 µM AHA during the 3 h recovery period.

Our BONCAT experiments (Figure 6c) revealed the profound impact of ivermectin and arsenite on de novo protein synthesis. Both ivermectin and arsenite significantly diminished the production of new proteins. The combination arsenite/ivermectin led to a particularly striking drop in translation. Since oxidants and other stressors induce Hsp70 synthesis [59], we monitored the production of new Hsp70 proteins. The side-by-side analysis of Hsp70 in SH-SY5Y and HeLa cells revealed that ivermectin profoundly impaired the synthesis of Hsp70 in neuroblastoma, but not in cervical carcinoma cells (Figure 6d). Published research demonstrates that Hsp70 is essential for SG disassembly during the recovery from stress induced by arsenite, heat shock, or proteasome inhibitors [43,46,60,61]. In particular, the inhibition of Hsp70 by VER-155008 impairs SG disassembly in both SH-SY5Y and HeLa cells [43,46,60,61]. This earlier research establishes a clear mechanistic link between Hsp70 inhibition and impaired SG disassembly in the cell lines used in our study.

We attempted to evaluate the effects of VER-155008 on SG dissolution in neuroblastoma and cervical carcinoma cells. Under our experimental conditions, the recovery of arsenite-treated cells in the presence of VER-155008 led to massive cell loss. These results are consistent with the observation that Hsp70 inhibition increases the vulnerability of cancer cells to stress [61]. Moreover, the data emphasize the essential role that Hsp70 plays for the recovery from stress.

In summary, BONCAT experiments uncovered a unique and prominent response to ivermectin when neuroblastoma cells recover from stress. We conclude that the loss of Hsp70 de novo synthesis in SH-SY5Y cells caused delayed SG clearance.

## 4. Discussion

Ivermectin and its derivatives are potential lead compounds for diverse clinical applications [1,5]. Many of the pertinent health conditions are linked to cell stress, including cancer and viral infections. Some of the proposed therapeutic interventions build on ivermectin’s ability to interfere with nuclear trafficking. This is relevant to our study, as several nuclear transport factors regulate granulostasis. However, none of the nuclear transport factors analyzed by us were candidate targets for the ivermectin-dependent delay of SG clearance in neuroblastoma cells.

SG composition and biological functions vary according to the stressor and cell type [13]. Furthermore, SG clearance is driven by the simultaneous action of multiple mechanisms [22]. The contributions of different pathways of SG removal depend on granule and cell properties. Our work underscores these observations, as SG clearance was especially sensitive to ivermectin in human neuroblastoma cells.

De novo synthesis of molecular chaperones is a mandatory step for cells to recover from various insults [59]. Inducible Hsp70s are particularly important to restore cellular homeostasis when stress subsides [58]. We have now identified Hsp70, a hub of the granulostasis network, as a key factor that is susceptible to modulation by ivermectin in SH-SY5Y cells. Our work revealed the remarkable deficit of new Hsp70 synthesis in neuroblastoma cells, despite the increase in *HSP70* transcripts. Several possible mechanisms could cause this discrepancy between transcript abundance and protein levels in SH-SY5Y cells. For example, ivermectin may affect translation factors that are particularly important in neuronal cells and relevant to stress responses. Possible candidates are eukaryotic elongation factor 1-α2 and mRNA-binding proteins [62,63]. Alternatively, ivermectin could modulate post-transcriptional modifications of *HSP70* mRNA in neuroblastoma cells and thereby affect its translation [64]. Our study was not designed to identify other transcripts whose translation is affected by ivermectin during the recovery from stress. This exciting possibility will be explored in the future.

A key question related to our research is the relevance for current and future applications of ivermectin in the clinic. Of particular interest are the ivermectin concentrations that can be achieved in the plasma or target tissues. While information on human subjects is less complete than for animal models, several general conclusions can be drawn for the treatment of mammals with ivermectin. First, peak concentrations of ivermectin in the plasma are in the nanomolar range [65,66]. More than 90% of ivermectin is protein-bound in the plasma [67]. Second, sex, nutritional state, and body mass index impact the pharmacokinetics of ivermectin [67]. Third, due to its lipophilic properties, ivermectin tends to accumulate in fat tissues [67]. Fourth, the peak concentration of ivermectin in plasma or target tissues depends on the route of administration [68]. At present, oral and topical applications are FDA-approved for humans [69].

Currently conducted or planned clinical trials assess the therapeutic potential of ivermectin, either alone or in combination with other compounds. Human diseases explored include COVID-19 and solid tumors [70]. Some of these trials plan to administer up to 400 µg/kg of ivermectin orally. However, data on the concentrations of ivermectin in plasma or target organs are not always available. In vitro studies commonly use ivermectin concentrations in the micromolar range, and we followed this approach. We did not measure the amount of ivermectin that was bound to serum in our experiments. Both cervical carcinoma and neuroblastoma cells were grown under identical conditions. Therefore, disparities in culturing conditions cannot explain the cell-context-specific differences we have uncovered for the effects of ivermectin.

To overcome the present limitations in the bioavailability of ivermectin, drug formulations are being developed and evaluated [71,72]. These strategies could increase the local or systemic concentrations of ivermectin, which will make our research particularly relevant to therapeutic application.

Taken together, the current study identified Hsp70 as a granulostasis factor for which de novo translation fails when SH-SY5Y cells attempt to regain homeostasis in the presence of ivermectin. Our results uncovered a divergence of mechanisms that promote stress recovery in human cancer cells. Moreover, we have shed light on the complex regulatory framework that shapes mammalian stress responses. Notably, we identified previously unknown effects of ivermectin on human cancer cells. The insights generated by us are particularly intriguing, as all stages of tumorigenesis, cancer progression, and metastasis rely on molecular chaperones [23]. While Hsp70 is a prime target for cancer therapy, the elimination of Hsp70 and its cancer-promoting activities remains difficult [73]. The present study adds a novel feature to the biology of heat shock proteins that can be explored to eradicate cancer cells. This avenue is especially promising, as many anticancer treatments induce SGs.

### Limitations of This Study

We identified Hsp70 as a critical component of the proteostasis network that can be derailed by ivermectin. However, we have not eliminated a possible contribution of granulophagy to SG clearance in SH-SY5Y or HeLa cells. Indeed, the effect of ivermectin on LC3β may support the idea that ivermectin slightly diminishes granulophagy in neuroblastoma cells.

It is noteworthy that non-toxic concentrations of ivermectin did not completely abolish SG clearance in either of the cell lines analyzed by us. This suggests that several mechanisms converge to remove SGs, and some of these pathways remain at least partially active in the presence of ivermectin.

Our research did not explore the possibility that ivermectin affects stress responses in neuroblastoma cells by activating receptors or channels [74]. These are open questions that should be addressed in future studies.

## Figures and Tables

**Table 1:** Sources of primary antibodies, dilutions for Western blotting and immunolocalization. NA, not applicable.

Primary Antibodies				
Protein	Supplier	Catalog Number	Dilution for Western Blotting	Dilution for Immunolocalization
Actin	Chemicon, Temecula, CA, USA	mab1501	1:100,000	NA
Bag3	Santa Cruz Biotechnology, Dallas, TX, USA	sc-136467	1:1000	NA
CAS	Santa Cruz Biotechnology	sc-271537	1:2000	NA
CHIP	Santa Cruz Biotechnology	sc-66830	1:1000	NA
Crm1 (*XPO1*)	Santa Cruz Biotechnology	sc-5595	1:1000	NA
HuR	Santa Cruz Biotechnology	sc-5261	1:2000	1:1000
p-eIF2α (S51)	ABclonal, Woburn, MA, USA	AP0745	1:1000	NA
eIF2α (total)	Santa Cruz Biotechnology	sc-30882	1:1000	NA
eIF2α (total)	ABclonal	A0764	1:1000	NA
p-G3BP1(S232)	ABclonal	AP0352	1:2000	NA
G3BP1 (total)	Santa Cruz Biotechnology	sc-365338	1:1000	1:500
Hsc70	Enzo Life Sciences, Toronto, ON, Canada	ADI-SPA-815	1:2000	NA
Hop (STIP1)	ABclonal	A14106	1:1000	NA
p-HSF1 (S326)	ABclonal	AP1140	1:1500	NA
HSF1 (total)	ABclonal	A13765	1:1500	1:200–1:500
Hsp40	Santa Cruz Biotechnology	sc-398766	1:2000	NA
Hsp70 (hsp72)	Enzo Life Sciences, Toronto, ON, Canada	ADI-SPA-812	1:2000	NA
Hsp70	Enzo Life Sciences	ADI-SPA-822	1:200	NA
Hsp90	Santa Cruz Biotechnology	sc-515081	1:1000	NA
Hsp110/Hsp105	Santa Cruz Biotechnology	sc-6241	1:2000	NA
HspBP1	Santa Cruz Biotechnology	sc-34252	1:2000	NA
Importin-α1 (*KPNA2*)	Santa Cruz Biotechnology	sc-5538	1:2000	NA
Importin-β1 (*KPNB1*)	Santa Cruz Biotechnology	sc-11367	1:1000	NA
LC3β	Santa Cruz Biotechnology	sc-376404	1:1000	NA
NFκB	Santa Cruz Biotechnology	sc-372	1:1000	NA
PP1, pan	Santa Cruz Biotechnology	sc-7482	1:2000	NA
p-PKR (T446)	abcam, Waltham, MA, USA	ab32036	1:2000	NA
PKR (total)	Gift from A. Gatignol, Montreal	NA	1:2000	NA
Ran	Santa Cruz Biotechnology	sc-1156	1:1000	NA
RanBP1	Santa Cruz Biotechnology	sc-28576	1:1000	NA
RCC1	Santa Cruz Biotechnology	sc-1161	1:1000	NA
TIA (TIA-1/TIAR)	ABclonal	A12523	1:1000	1:250
Transportin 1/karyopherin-β2 (*TPNO1*)	Santa Cruz Biotechnology	sc-166127	1:2000	NA
VCP	BioLegend, San Diego, CA, USA	636802	1:1000	NA
**Secondary Antibodies**				
**Tag**		**Supplier**	**Dilution for** **Western Blotting**	**Dilution for** **Immunolocalization**
Horseradish peroxidase (HRP)		Jackson ImmunoResearch, West Grove, PA, USA	1:2000	NA
Alexa Fluor^®^ 488, Cy3		Jackson ImmunoResearch	NA	1:200–1:500

**Table 2:** Primers for qPCR.

Target Gene	Forward Sequence	Reverse Sequence
*HSPA1A*	5′-ACCTTCGACGTGTCCATCCTGA-3′	5′-TCCTCCACGAAGTGGTTCACCA-3′
*HSPH1*	5′-ACTGCTTGTTCAAGAGGGCTGTGA-3′	5′-AACATCCACACCCACACACATGCT-3′
*ACTB*	5′-ACCATTGGCAATGAGCGGTTC-3′	5′-AGGTCTTTGCGGATGTCCACGT-3′

## Data Availability

The original contributions presented in this study are included in the article/Supplementary Material. Further inquiries can be directed to the corresponding author.

## Supplementary Materials

The following supporting information can be downloaded at: https://www.mdpi.com/article/10.3390/cells15171623/s1, Figure S1: Ivermectin toxicity in SH-SY5Y and HeLa cells. Figure S2: Ivermectin inhibits classical nuclear import. Figure S3: Ivermectin alters stress signaling in a cell context dependent fashion. Figure S4: Comparison of stress signaling components in neuroblastoma and cervical carcinoma cells. Figure S5: The phosphorylation and abundance of nucleating SG proteins differ in SH-SY5Y and HeLa cells. Figure S6: Impact of ivermectin on the abundance of nuclear transport factors. Figure S7: Comparison of the abundance of nuclear transport factors in neuroblastoma and cervical carcinoma cells. Figure S8: Effects of ivermectin and cellular context on LC3β, a key component of the autophagy-lysosomal pathway. Figure S9: SH-SY5Y and HeLa cells differ in the abundance of molecular chaperones that are relevant to SG clearance. Figure S10: Different antibodies confirm that HspB8 abundance is low in neuroblastoma compared with cervical carcinoma cells. Figure S11: Hsf1 phosphorylation and abundance in SH-SY5Y and HeLa cells under control and oxidative stress conditions. Figure S12: Original Western blots related to Figure 3a and Figure S4. Figure S13: Original Western blots related to Figure 3b. Figure S14: Original Western blots related to Figure S5. Figure S15: Original Western blots related to Figure S6. Figure S16: Original Western blots related to Figure S7. Figure S17: Original Western blots related to Figure S8. Figure S18: Original Western blots related to Figure 4; part 1. Figure S19: Original Western blots related to Figure 4; part 2. Figure S20: Original Western blots related to Figure S9. Figure S21: Original Western blots related to Figure S10. Figure S22: Original Western blots related to Figure 5a. Figure S23: Original Western blots related to Figure S11. Figure S24: Original Western blots related to Figure 6c,d.

## Abbreviations

The following abbreviations are used in this manuscript:

AHA	L-azidohomoalanine
ANOVA	Analysis of Variance
BCS	bovine calf serum
BONCAT	Bioorthogonal Non-Canonical Amino Acid Tagging
BSA	bovine serum albumin
DAPI	4′,6-diamidino-2-phenylindole
ECL	enhanced chemiluminescence
G3BP1	Ras-GTPase-Activating Protein SH3-Domain-Binding Protein 1
HRP	Horseradish peroxidase
NLS	nuclear localization sequence
PP1	Phosphoprotein phosphatase 1
PP2A	Phosphoprotein phosphatase 2 A
PKR	protein kinase R
SEM	standard error of the mean
SG	stress granule
