# Supplementary material for: Ivermectin Inhibits Stress Granule Clearance by Blocking the De Novo Synthesis of Hsp70 in Neuroblastoma Cells
Source: Cells. 2026 Sep 7;15(17):1623. doi: 10.3390/cells15171623 (PMC13564725; doi:10.3390/cells15171623)
Supplement: Supplementary file 1 [file cells-15-01623-s001.zip › cells-4068139-supplementary.pdf]

Supplementary File

Ivermectin inhibits stress granule clearance by blocking the *de novo* synthesis of Hsp70 in neuroblastoma cells

Siwei Chu[1], Elizabeth P. Anim[1], Reyhaneh Salehi-Tabar[1], John H. White[1] and Ursula Stochaj[1, 2,*]

[1]Department of Physiology, McGill University, Montreal, Canada; [2]Quantitative Life Sciences Program, McGill University, Montreal, Canada

[*]Correspondence to: ursula.stochaj@mcgill.ca

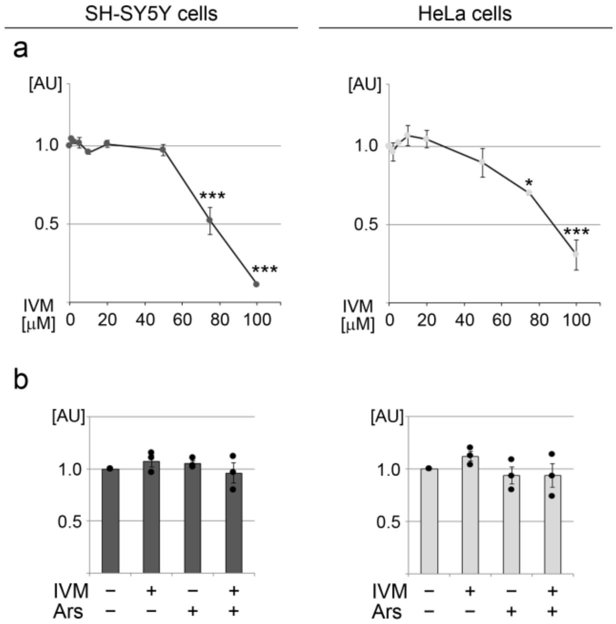

Figure S1. Ivermectin toxicity in SH-SY5Y and HeLa cells. The cell viability was determined for n=3 independent experiments for the conditions shown. Data were normalized to the control conditions. Graphs depict averages ± SEM (standard error of the mean). Significant differences were identified by One-way ANOVA combined with Bonferroni post hoc correction. *, $p < 0.05$; ***, $p < 0.001$. AU, arbitrary units. (a) Cultured cells were exposed for 24h to increasing concentrations of ivermectin (IVM). Cell viability was measured at the end of the incubation period. (b) Cells were treated with vehicle or 0.5mM sodium arsenite (Ars) for 30min, followed by stress recovery without or with 50µM ivermectin for 3h. Cell viability was determined after 3h recovery. AU, arbitrary units.

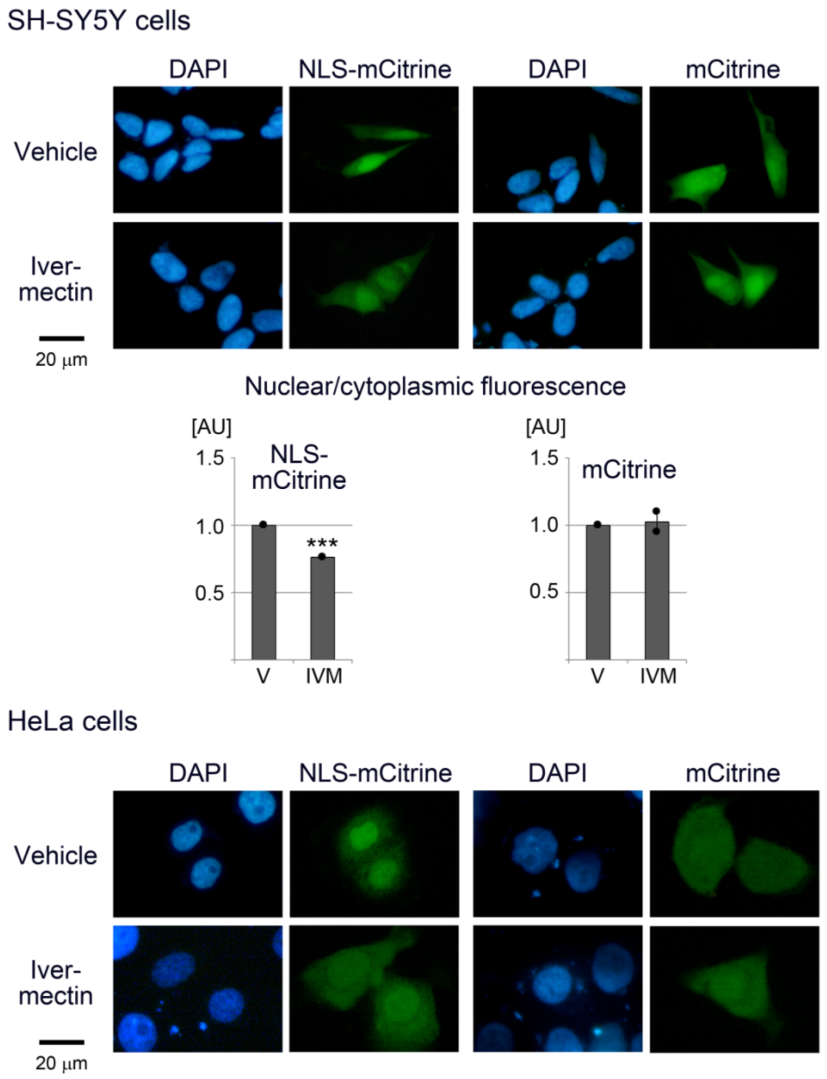

Figure S2. Ivermectin inhibits classical nuclear import. SH-SY5Y or HeLa cells were transfected with plasmids encoding SV40-NLS fused to mCitrine (NLS-mCitrine) or coding for mCitrine. The nuclear/cytoplasmic distribution of fluorescence was quantified for SH-SY5Y cells incubated with vehicle (V) or ivermectin (IVM). Data are shown for n=2 independent experiments. For each experiment and condition, 18 to 21 cells were evaluated. Student's t-test identified significant differences between vehicle and ivermectin-treated cells; ***, $p < 0.001$. AU, arbitrary units. In HeLa cells, ivermectin markedly diminished the nuclear accumulation of NLS-mCitrine. Size bar is 20µm.

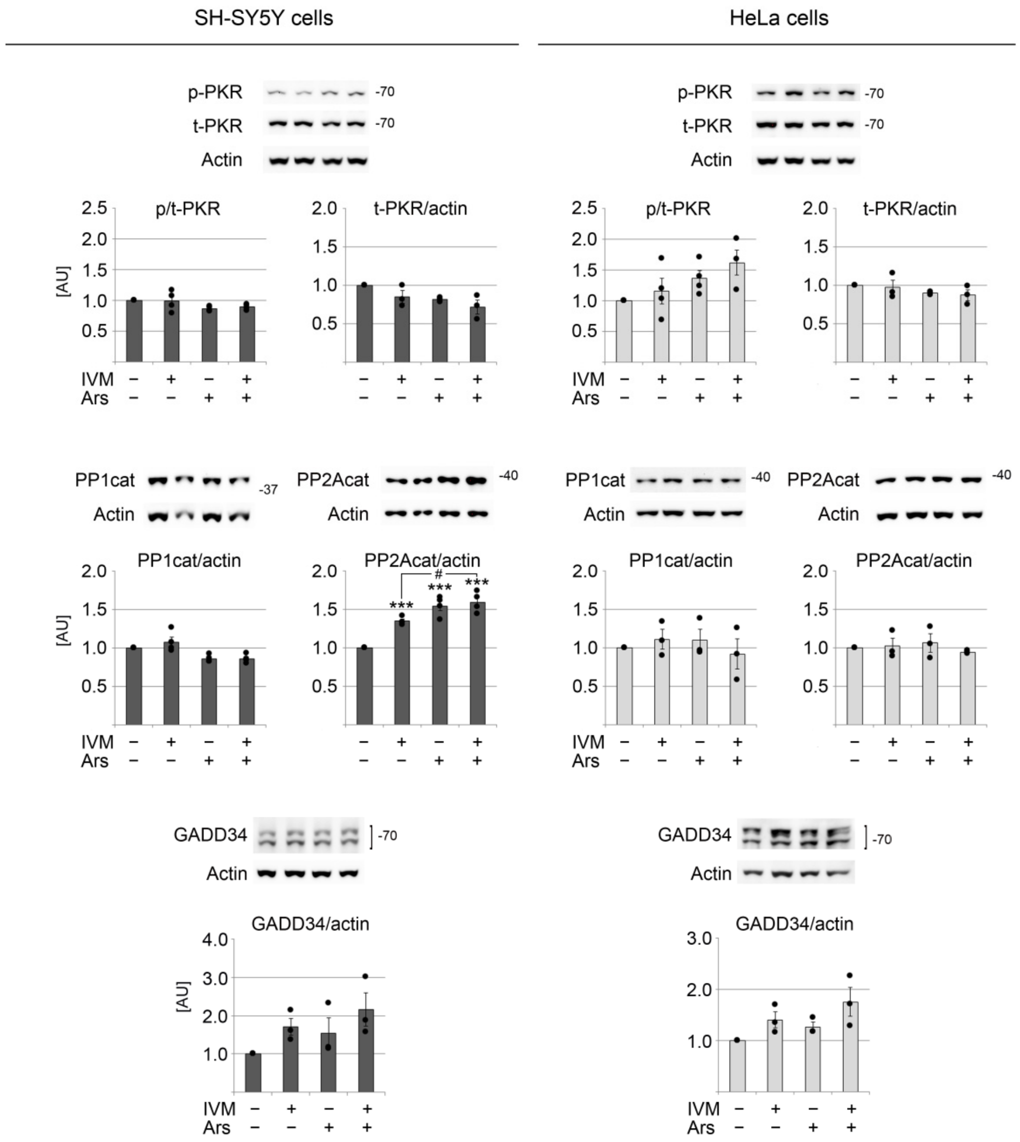

Figure S3. Ivermectin alters stress signaling in a cell context dependent fashion. SH-SY5Y and HeLa cells were incubated with vehicle or arsenite (Ars) to generate SGs. During the subsequent 3h stress recovery vehicle (-IVM) or ivermectin (+IVM) was present. Stress-related signaling events were quantified for at least three (n≥3) independent experiments. Results were normalized to the vehicle control (-IVM, -Ars). Graphs depict averages ± SEM as arbitrary units [AU]. Statistically significant differences were determined by One-way ANOVA plus Bonferroni post hoc correction. Comparisons were made pairwise to the control (-IVM, -Ars); ***, $p < 0.001$ or between treatment groups #, $p < 0.05$.

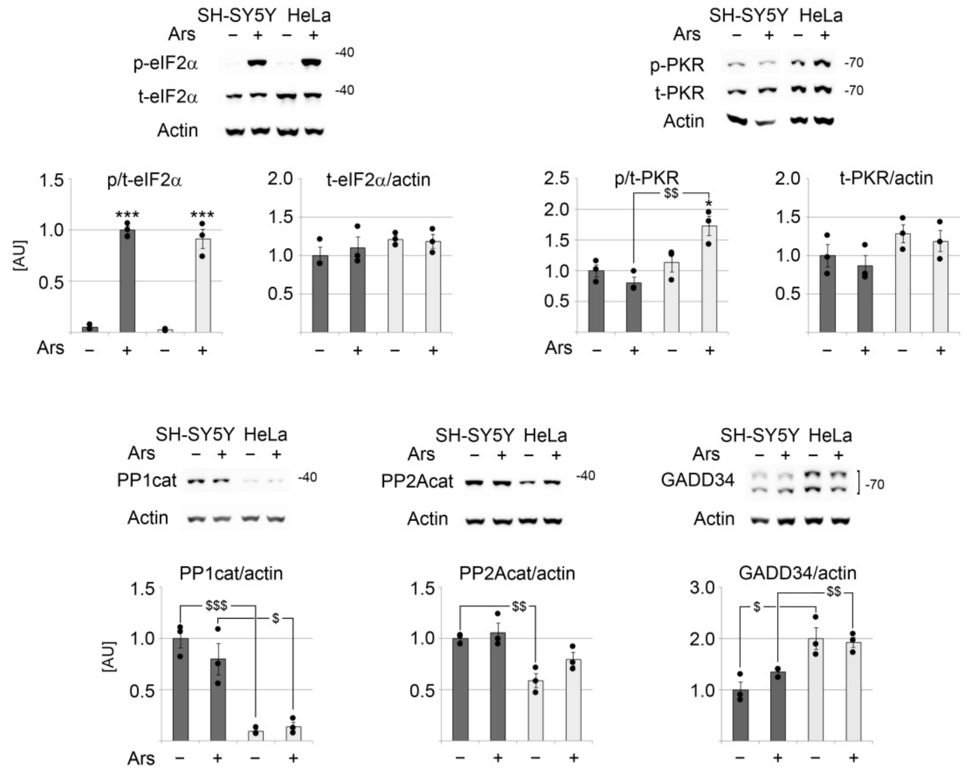

Figure S4. Comparison of stress signaling components in neuroblastoma and cervical carcinoma cells. Crude extracts were prepared for SH-SY5Y and HeLa cells incubated with vehicle or arsenite (30 min 0.5mM arsenite), as indicated. Cell extracts were assessed for eIF2α and PKR phosphorylation and the abundance of signaling factors. The treatment of cells, processing of samples and Western blotting were carried out as described for Fig. 3a. Graphs depict the results for at least n=3 independent experiments. All results are shown as arbitrary units, AU. Significant differences between vehicle and arsenite treatment were identified by Student's t-test. ***, $p < 0.001$. Student's t-test was also used to compare data for SH-SY5Y and HeLa cells. $, $p < 0.05$; $$, $p < 0.01$; $$$, $p < 0.001$.

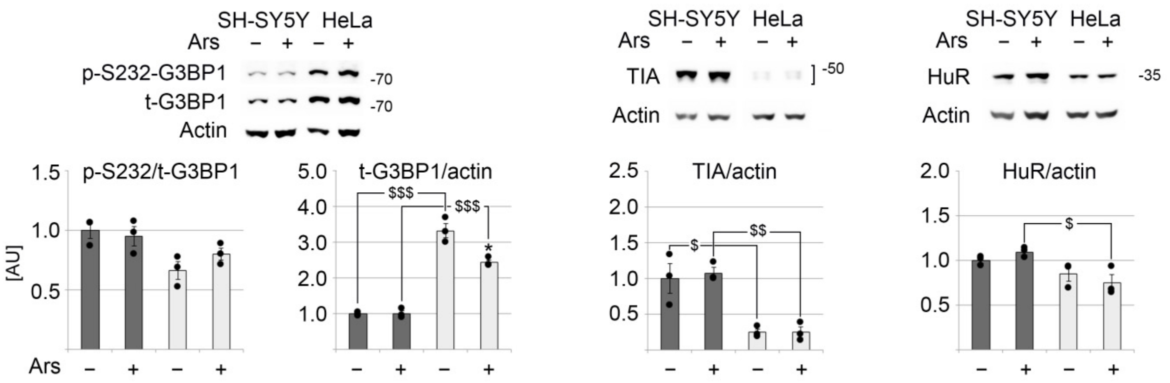

Figure S5. The phosphorylation and abundance of nucleating SG proteins differ in SH-SY5Y and HeLa cells. Crude cell extracts were examined by Western blotting for the phosphorylation of G3BP1 on S232. The abundance of total G3BP1 (t-G3BP1), TIA and HuR were measured as well. The treatment of cells, processing of samples and Western blotting were carried out as described for Fig. 3a. Data are shown for at least n=3 independent experiments. Student's t-test identified significant differences between SH-SY5Y and HeLa cells. \$, $p < 0.05$; \$\$, $p < 0.01$; \$\$\$, $p < 0.001$. AU, arbitrary units.

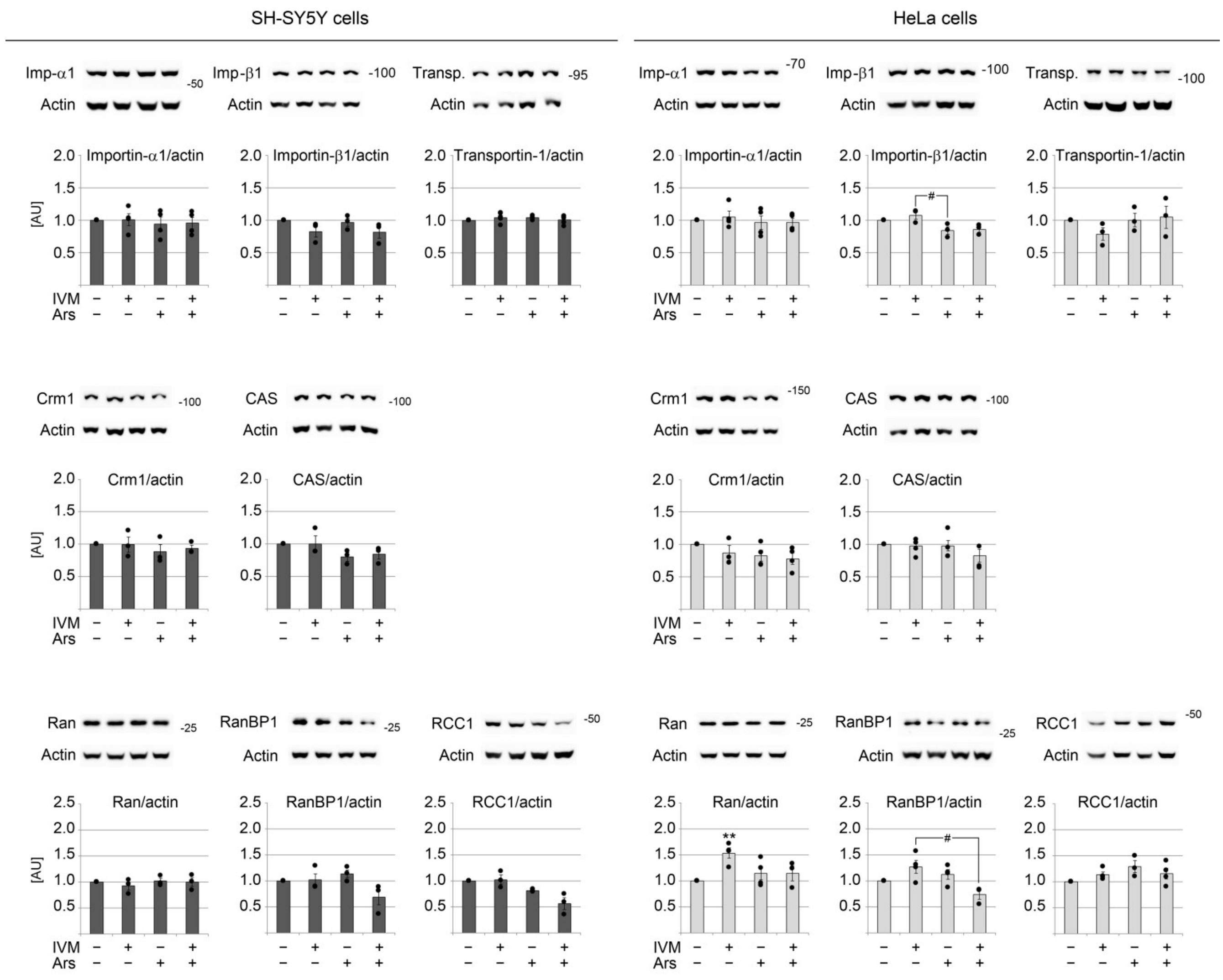

Figure S6. Impact of ivermectin on the abundance of nuclear transport factors. Different nuclear transport factors were evaluated by Western blot analyses, as described for Fig. 3a. Each data point represents at least n=3 independent experiments. Statistical evaluation was performed with One-Way ANOVA combined with Bonferroni post hoc analysis. Comparison relative to control sample (-IVM, -Ars): **, $p < 0.01$; simultaneous comparison of all pairs: #, $p < 0.05$. Imp-β1, Importin-β1; Transp., Transportin-1. AU, arbitrary units.

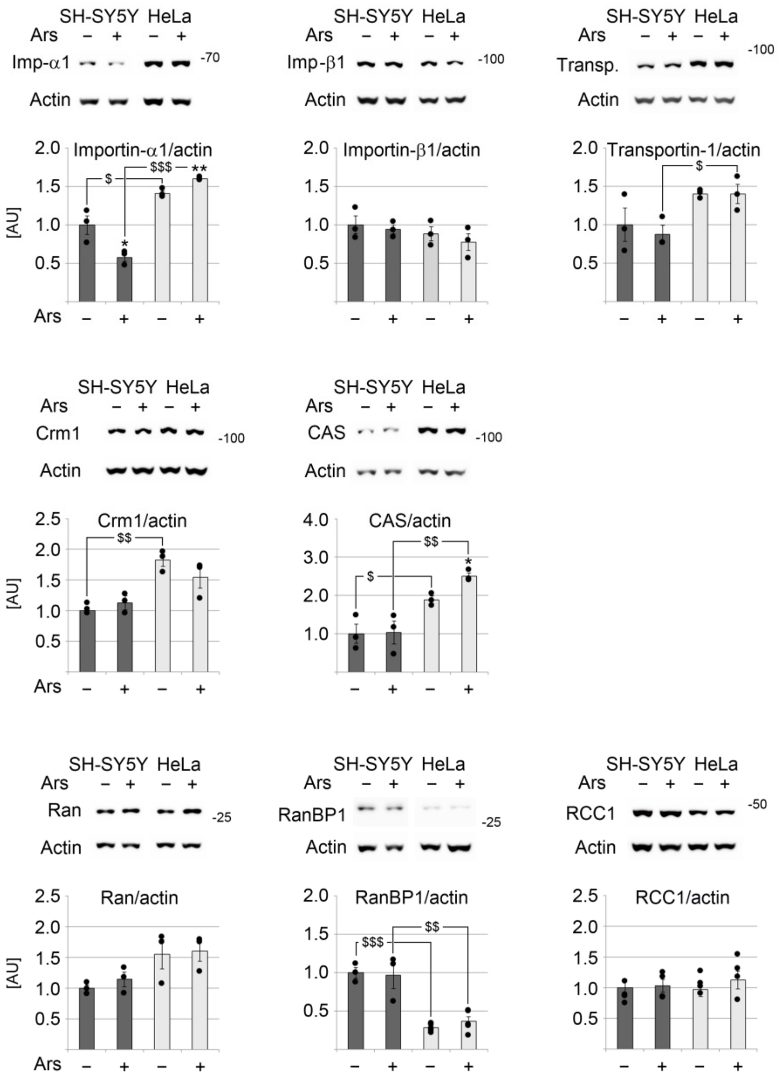

Figure S7. Comparison of the abundance of nuclear transport factors in neuroblastoma and cervical carcinoma cells. Crude extracts prepared for SH-SY5Y and HeLa were assessed by Western blotting as described for Fig. 3a. Graphs depict average ±SEM for a minimum of n=3 independent experiments. Student's t-test identified significant differences between vehicle and arsenite-treated SH-SY5Y or HeLa cells. *, $p < 0.05$; **, $p < 0.01$. Student's t-test also evaluated the differences between SH-SY5Y and HeLa cells. $, $p < 0.05$; $$, $p < 0.01$; $$$, $p < 0.001$. AU, arbitrary units.

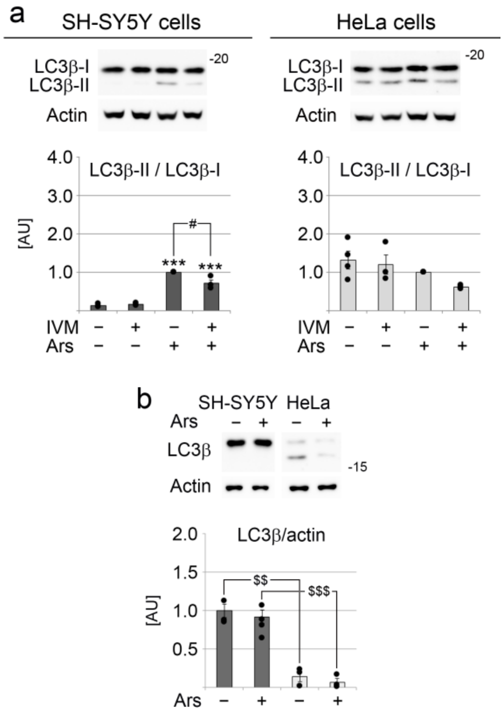

Figure S8. Effects of ivermectin and cellular context on LC3β, a key component of the autophagy-lysosomal pathway. The preparation of cell extracts and Western blotting were performed as described for Fig. 3a. At least n=3 independent experiments were conducted for each dataset. (a) Examination of LC3-β-I and LC3-β-II in stressed and ivermectin-treated cells. Statistical evaluation was carried out by One-Way ANOVA and Bonferroni post hoc analysis. Pairwise comparison to controls (-IVM, -Ars), ***, $p < 0.001$; pairwise comparison between treatment groups, # $p < 0.05$. (b) Comparison of LC3β levels in SH-SY5Y and HeLa cells. Significant differences were identified with Students t-test. $$, $p < 0.01$; $$$, $p < 0.001$. AU, arbitrary units.

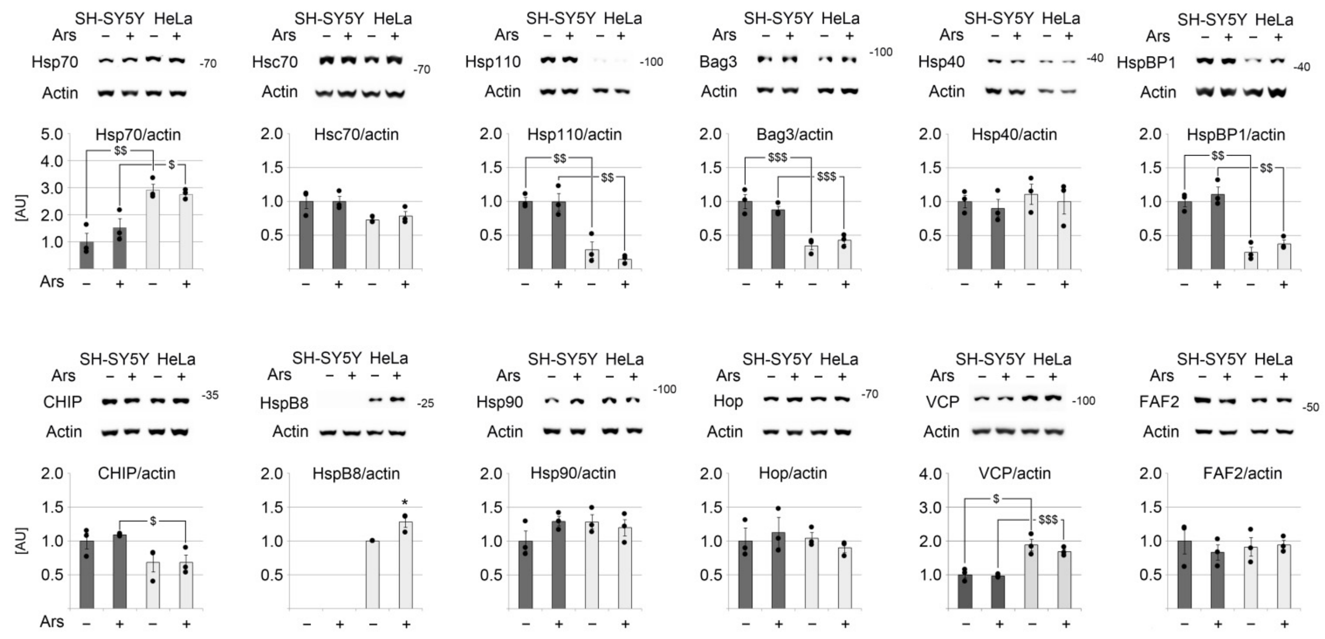

Figure S9. SH-SY5Y and HeLa cells differ in the abundance of molecular chaperones that are relevant to SG clearance. SH-SY5Y and HeLa cells were incubated with vehicle or 0.5mM arsenite for 30min. Crude extracts were used for Western blotting with antibodies against molecular chaperones and their co-factors, using the methods described for Fig. 3a. The molecular mass of marker proteins in kD is indicated on the right side of the blot. Graphs depict the results ± SEM for at least n=3 independent experiments. Data were normalized to the vehicle control of SH-SY5Y cells. Results for neuroblastoma and cervical carcinoma cells were compared with Student's t-test. \$, $p < 0.05$; \$\$, $p < 0.01$; \$\$\$, $p < 0.001$. The low abundance of HspB8 in SH-SY5Y compared with HeLa cells was confirmed with a different antibody (Fig. S10). AU, arbitrary units.

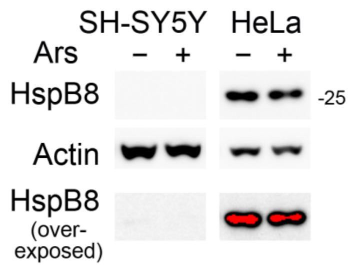

Figure S10. Different antibodies confirm that HspB8 abundance is low in neuroblastoma compared with cervical carcinoma cells. Crude extracts were prepared for SH-SY5Y and HeLa cells incubated in the absence or presence of arsenite for 30min. Samples were analyzed by Western blotting with a polyclonal antibody against HspB8. All samples were on the same blot with identical exposure times. The position of the 25kD marker protein is depicted at the right margin of the blot. Note that a different antibody was used to detect HspB8 in Fig. 2. The ECL signal for HspB8 in SH-SY5Y cells was faint even after prolonged exposure ("overexposed").

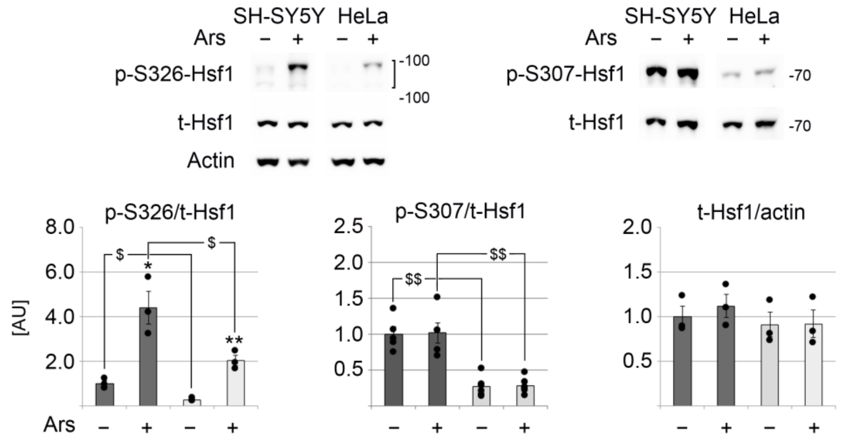

Figure S11. Hsf1 phosphorylation and abundance in SH-SY5Y and HeLa cells under control and oxidative stress conditions. The treatment of cultured cells, preparation of crude extracts, Western blotting, and statistical evaluation were conducted as described for Fig. 3a. Student's t-test identified significant differences between vehicle controls and arsenite treated cells. *, $p < 0.05$; **, $p < 0.01$. Student's t-test was also used to compare SH-SY5Y with HeLa cells. \$, $p < 0.05$; \$\$, $p < 0.01$. AU, arbitrary units.

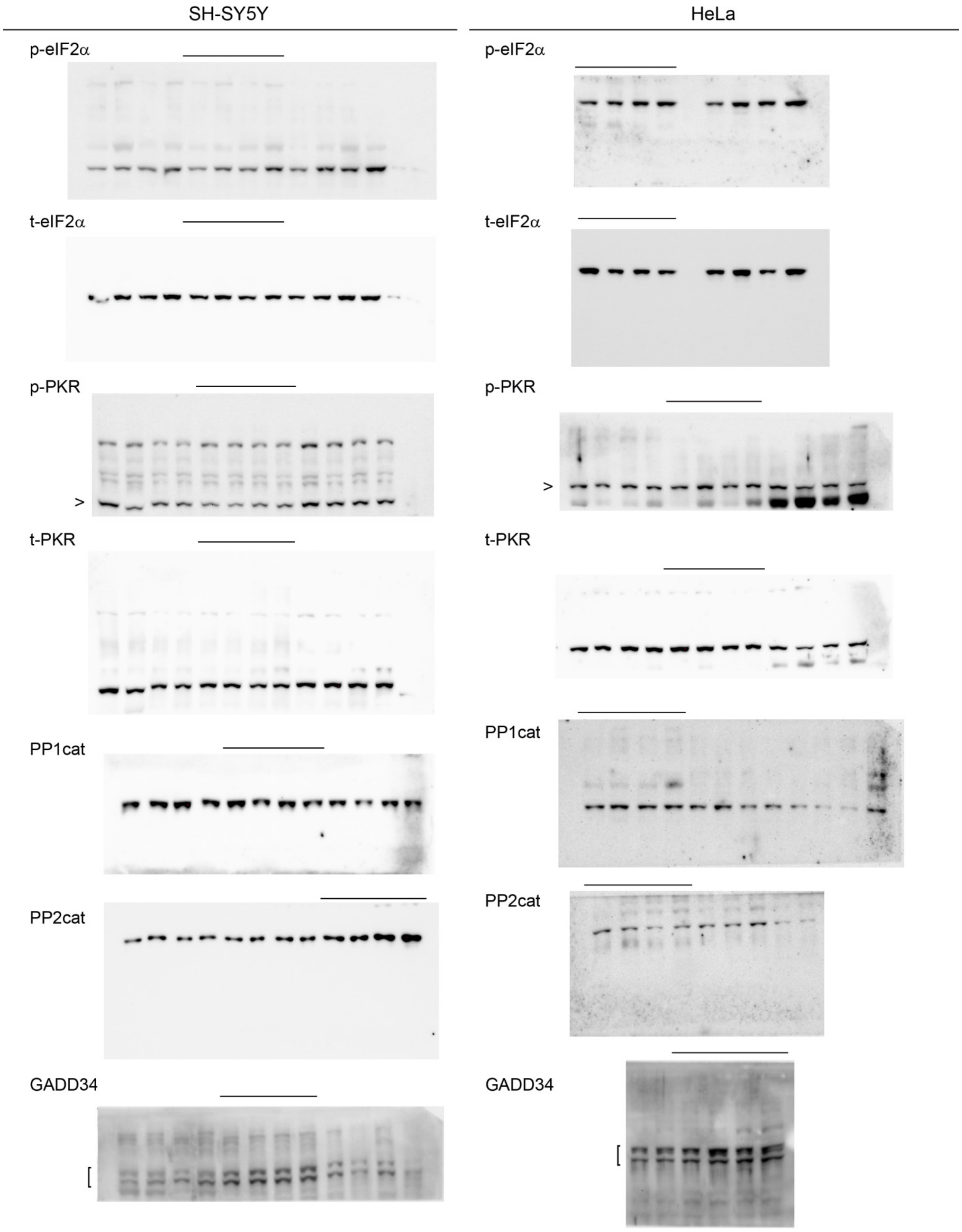

Figure S12. Original Western blots related to Figure 3a and S4. Vertical lines above the blot indicate the lanes included in the figure. Positions of the bands of interest are indicated with arrowheads (>) or brackets ([).

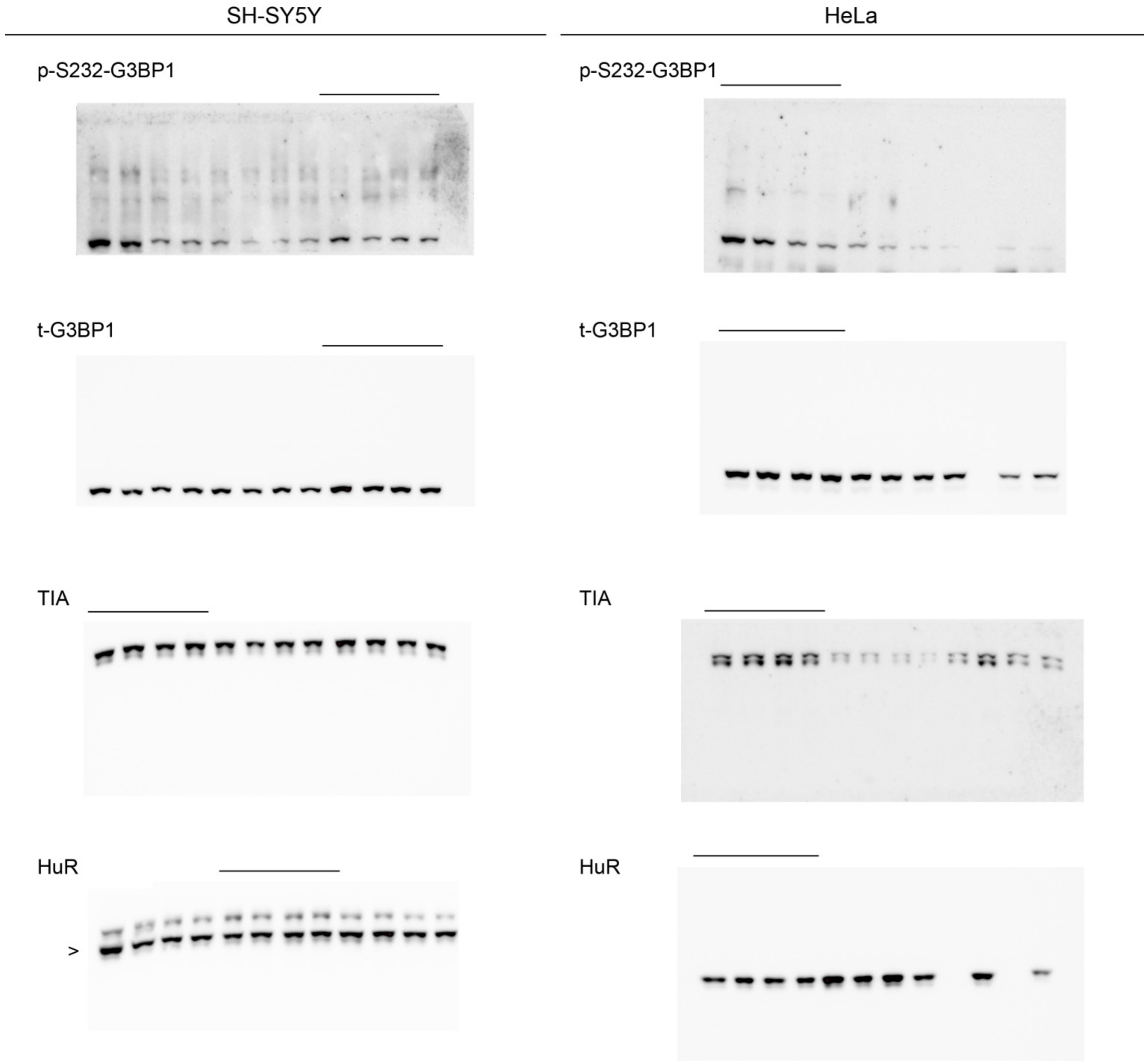

Figure S13. Original Western blots related to Figure 3b. Vertical lines above the blot indicate the lanes included in the figure. The position of HuR is marked by an arrowhead (>).

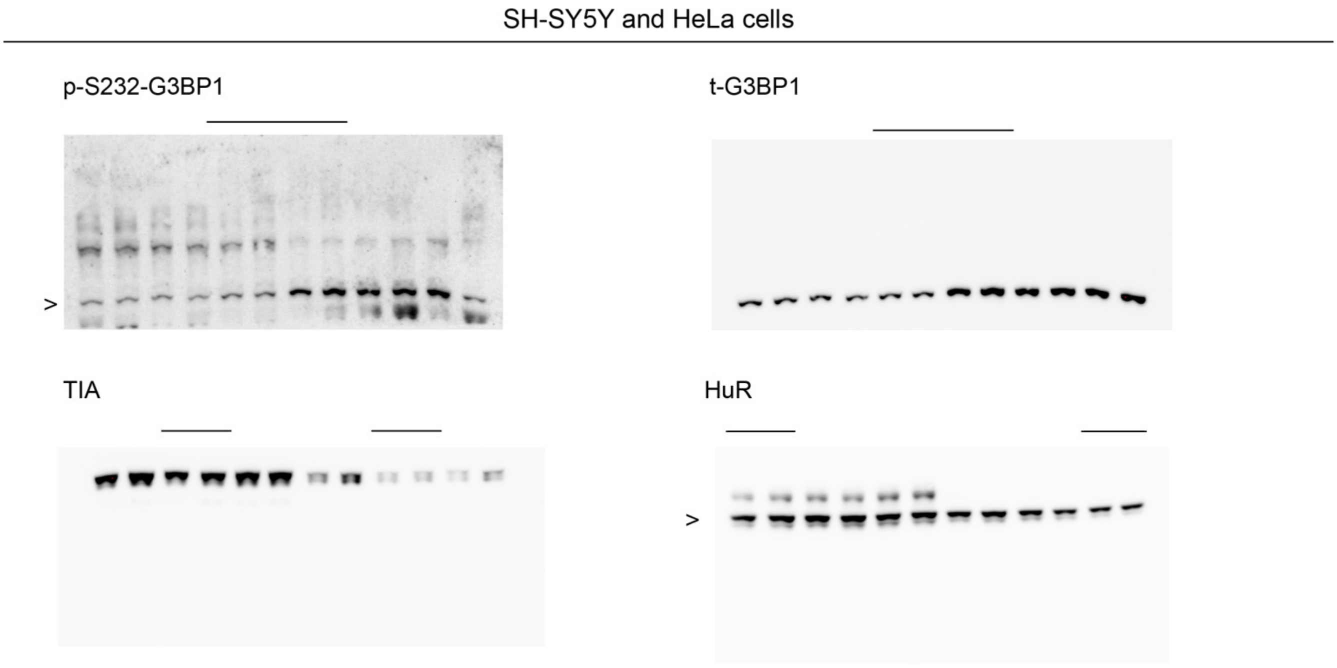

Figure S14. Original Western blots related to Figure S5. Vertical lines above the blot indicate the lanes included in the figure. The position of HuR is marked by an arrowhead (>).

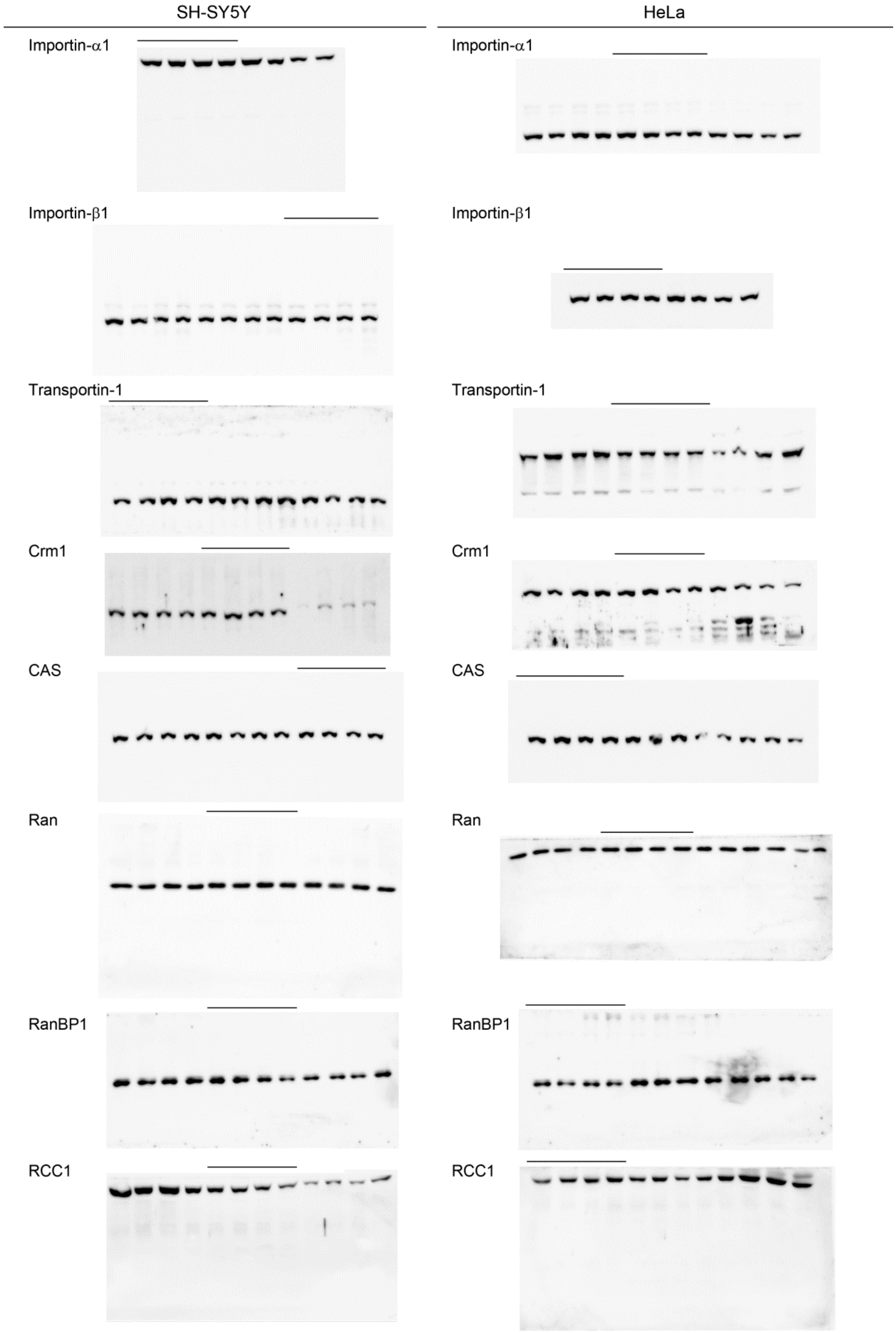

Figure S15. Original Western blots related to Figure S6. Vertical lines above the blot indicate the lanes included in the figure.

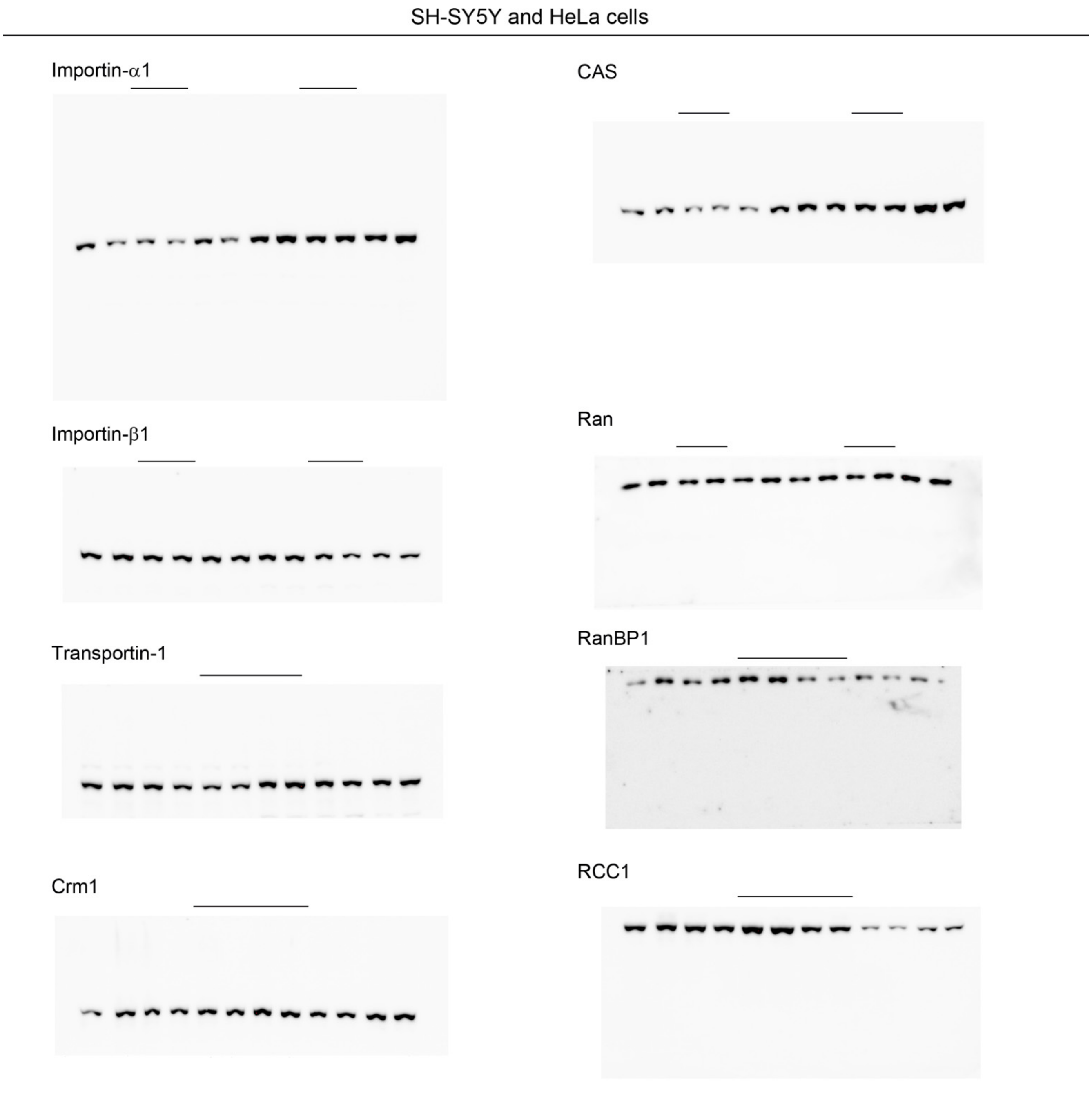

Figure S16. Original Western blots related to Figure S7. Vertical lines above the blot indicate the lanes included in the figure.

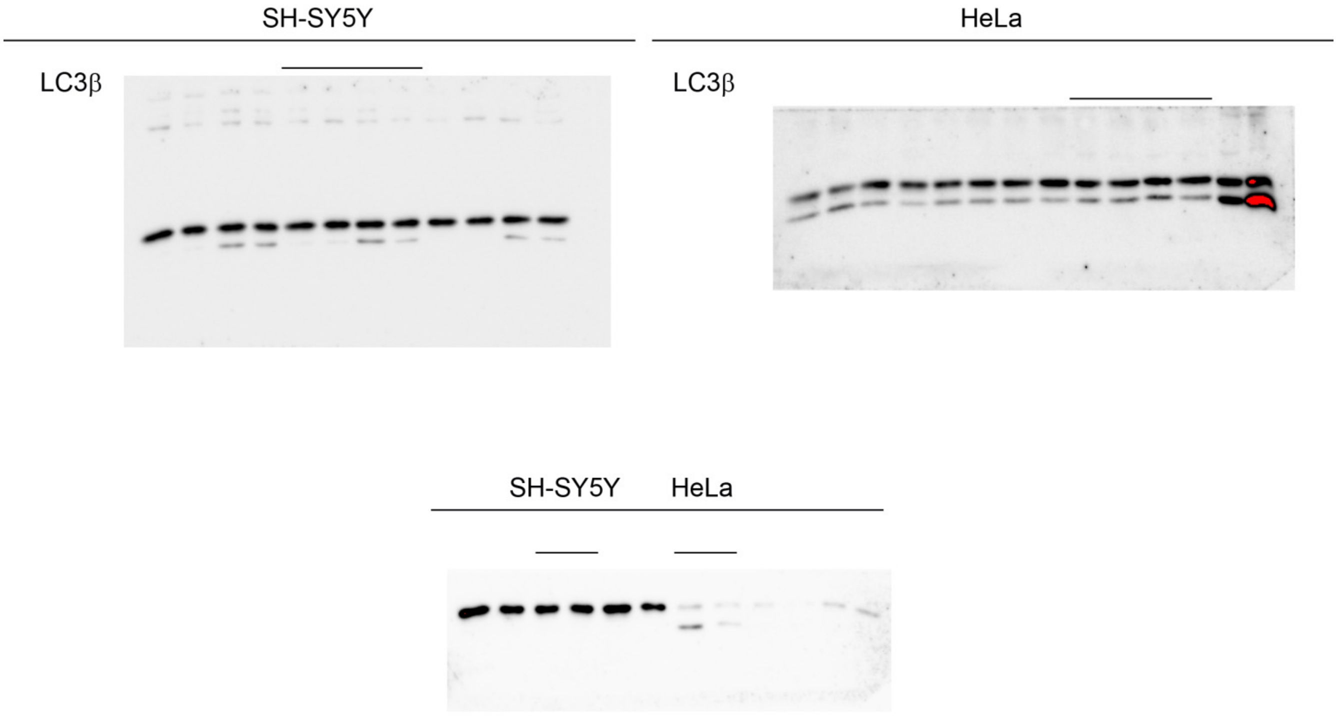

Figure S17. Original Western blots related to Figure S8. Vertical lines above the blot indicate the lanes included in the figure.

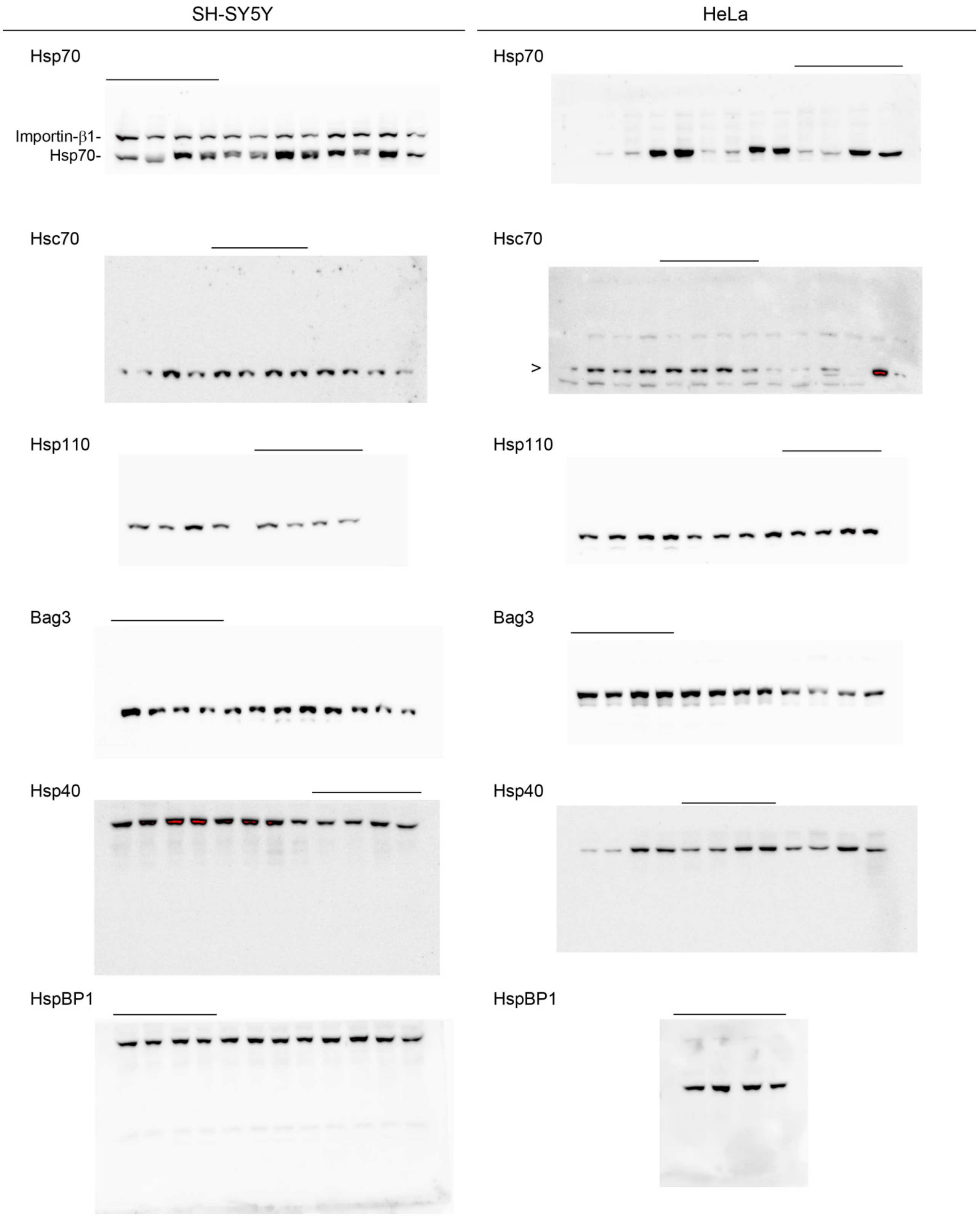

Figure S18. Original Western blots related to Figure 4. Part 1 of original blots. Vertical lines above the blot indicate the lanes included in the figure.

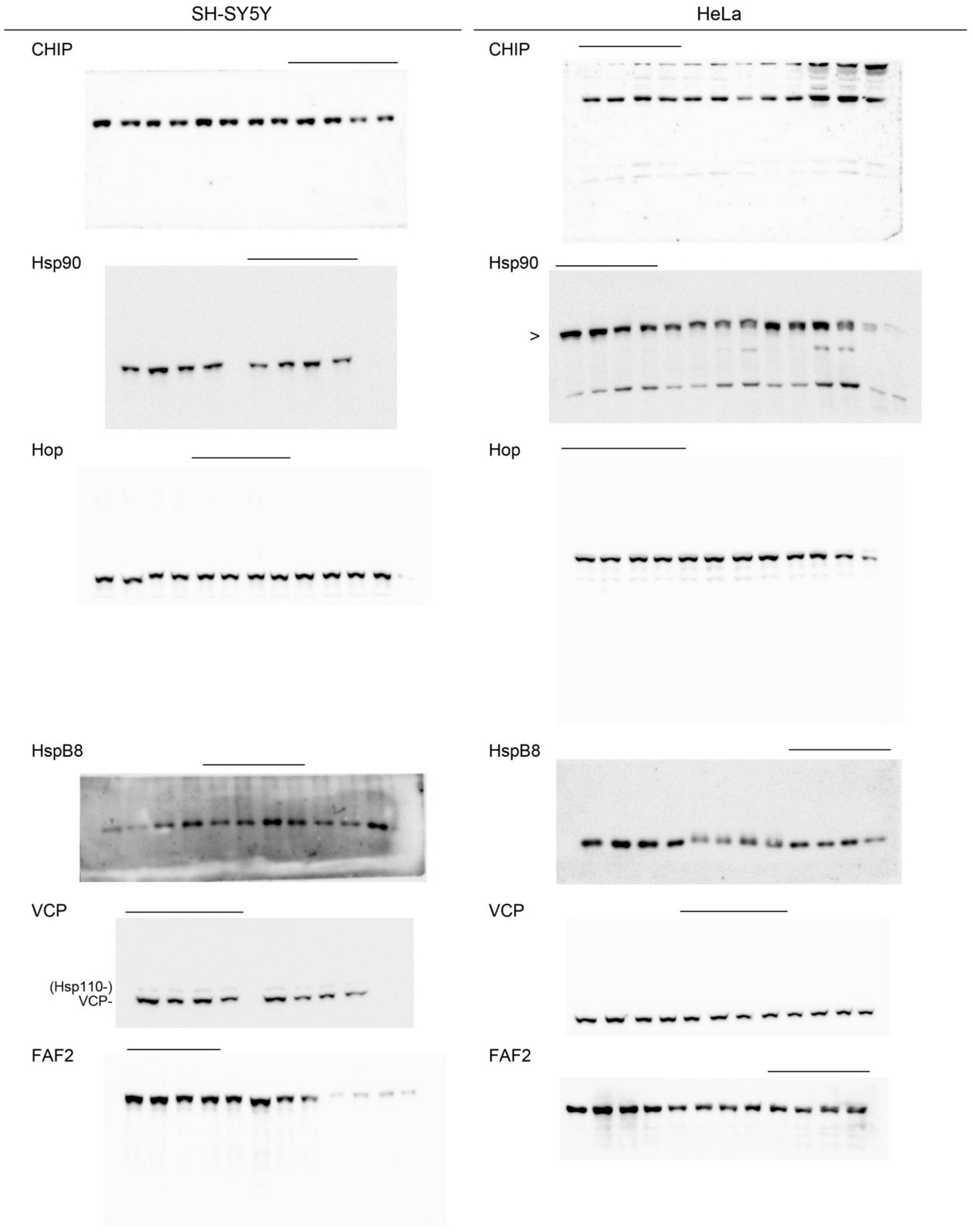

Figure S19. Original Western blots related to Figure 4. Part 2 of original blots. Vertical lines above the blot indicate the lanes included in the figure. The position of Hsp90 is marked by an arrowhead (>), and the position of VCP is shown for samples from SH-SY5Y cells.

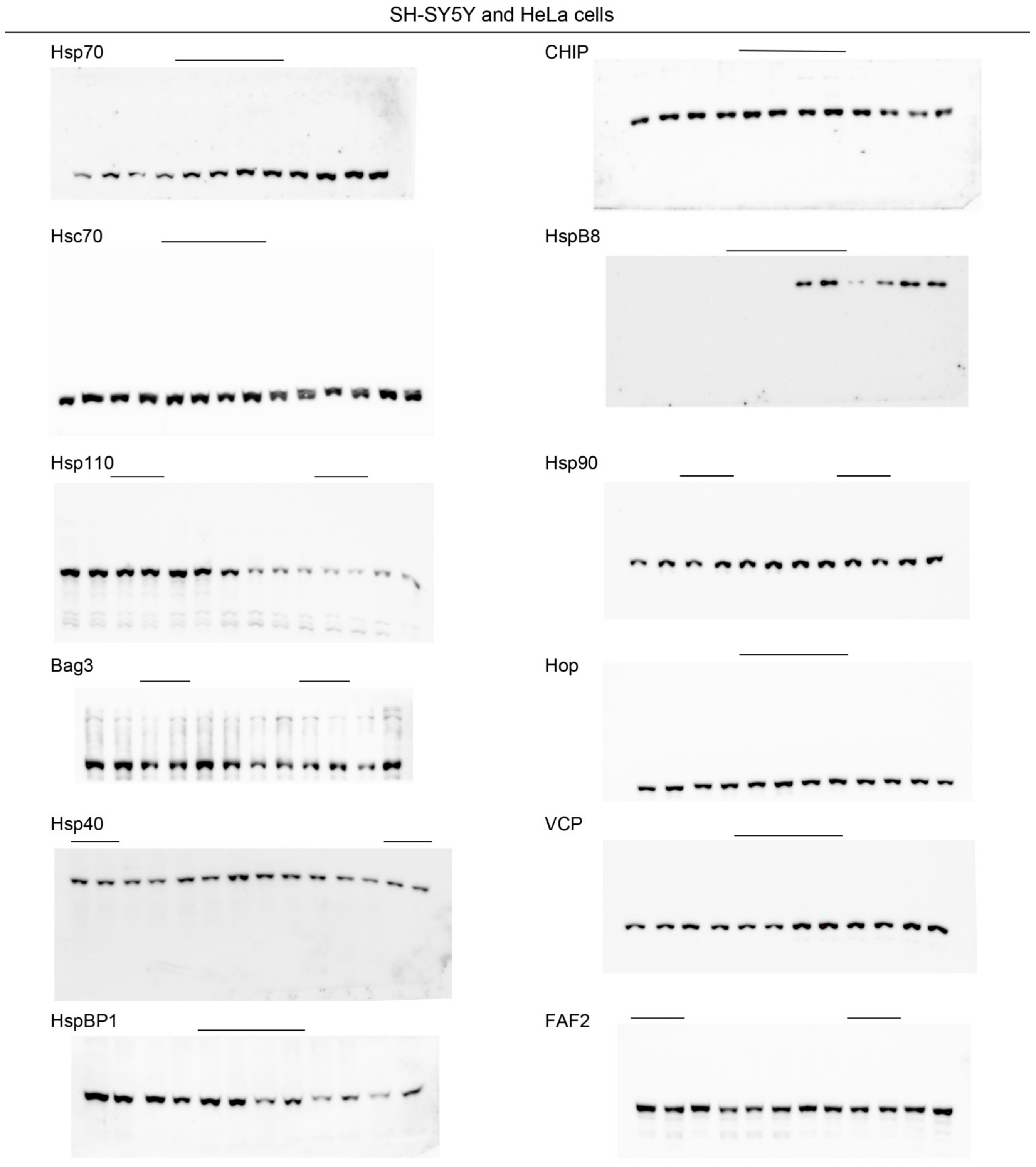

Figure S20. Original Western blots related to Figure S9. Vertical lines above the blot indicate the lanes included in the figure.

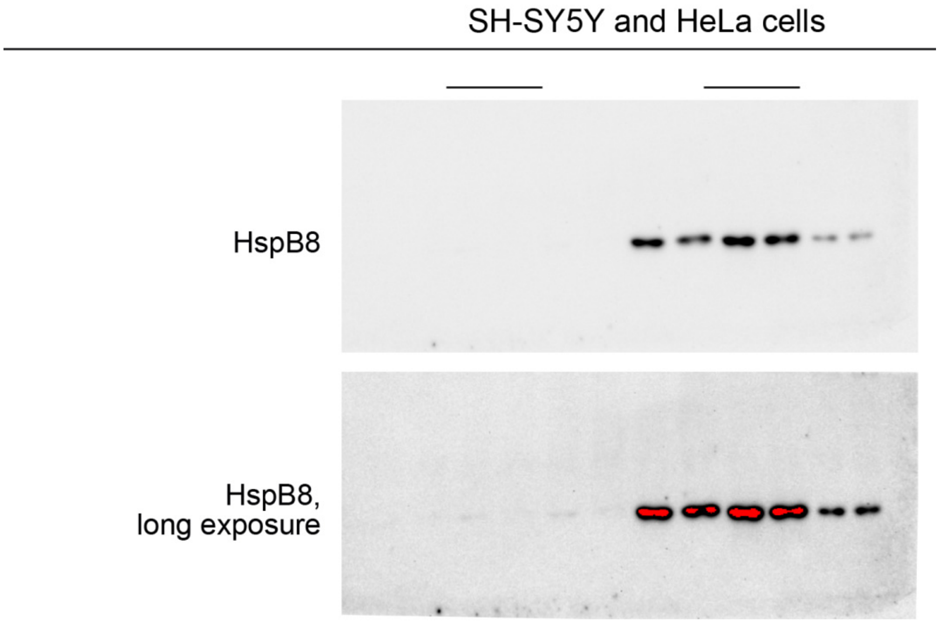

Figure S21. Original Western blots related to Figure S10. Vertical lines above the blot indicate the lanes included in the figure.

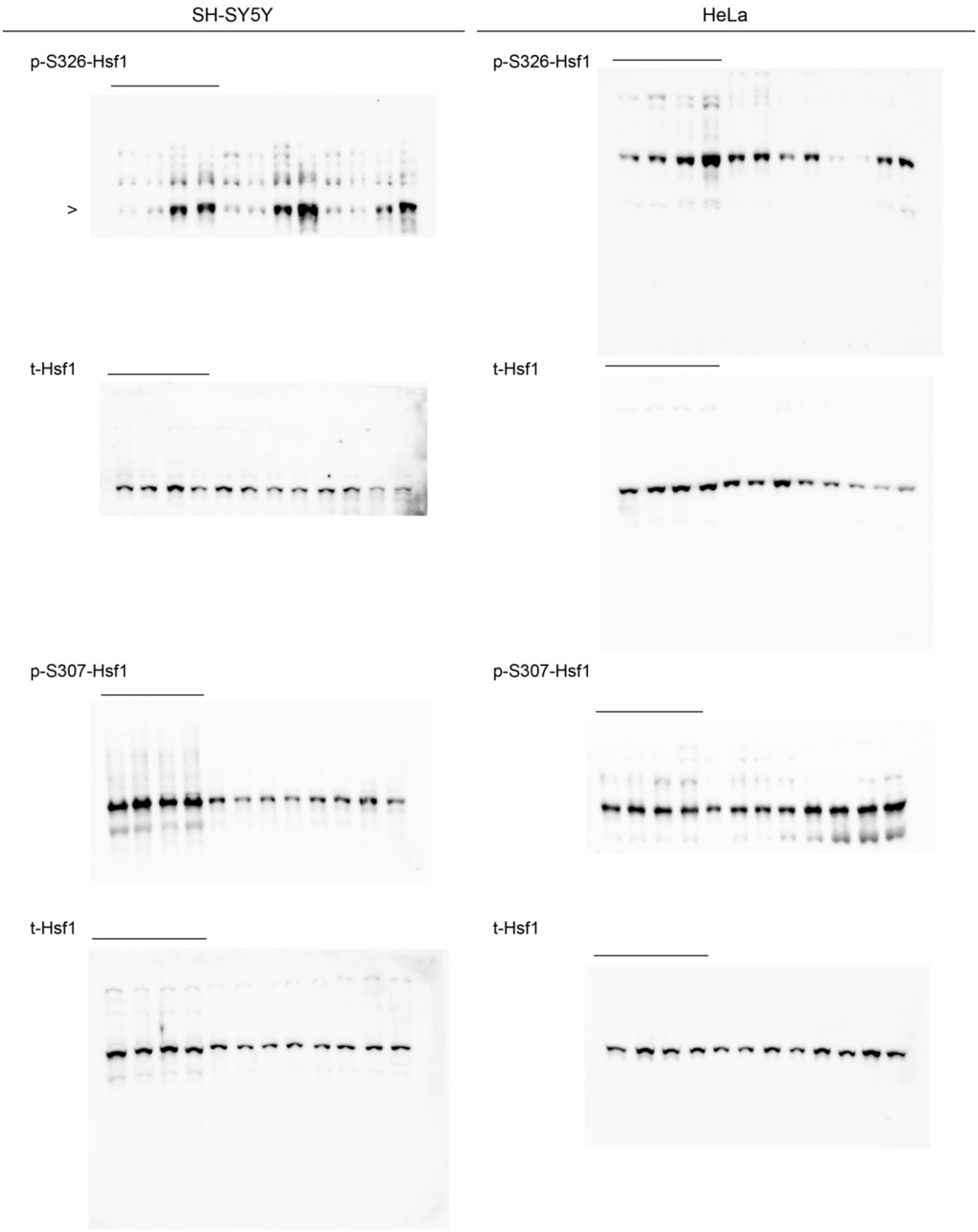

Figure S22. Original Western blots related to Figure 5a. Vertical lines above the blot indicate the lanes included in the figure. The position of p-S326-Hsf1 for SH-SY5Y cell extracts is marked by an arrowhead (>).

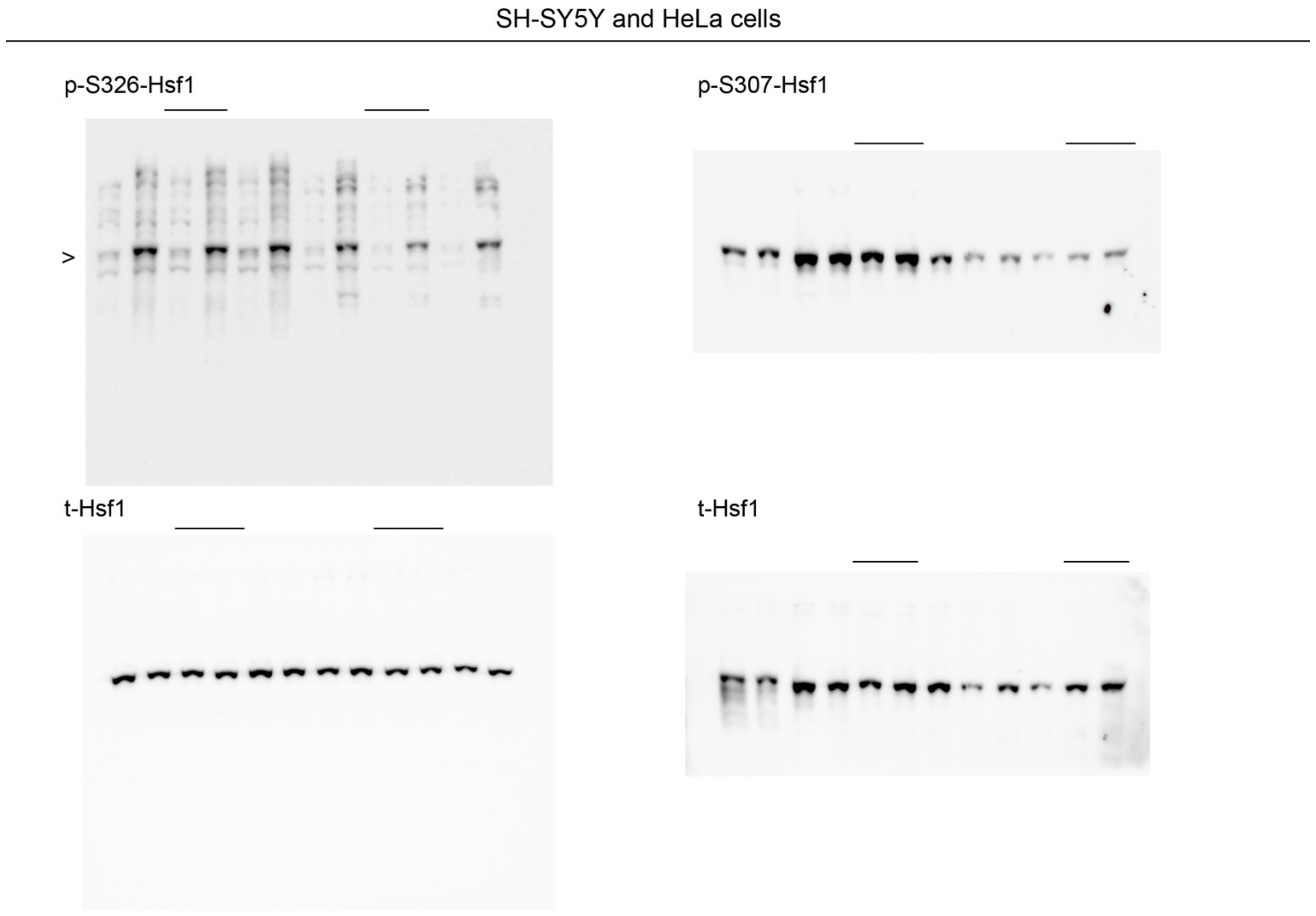

Figure S23. Original Western blots related to Figure S11. Vertical lines above the blot indicate the lanes included in the figure. The position of p-S326-Hsf1 is marked by an arrowhead (>).

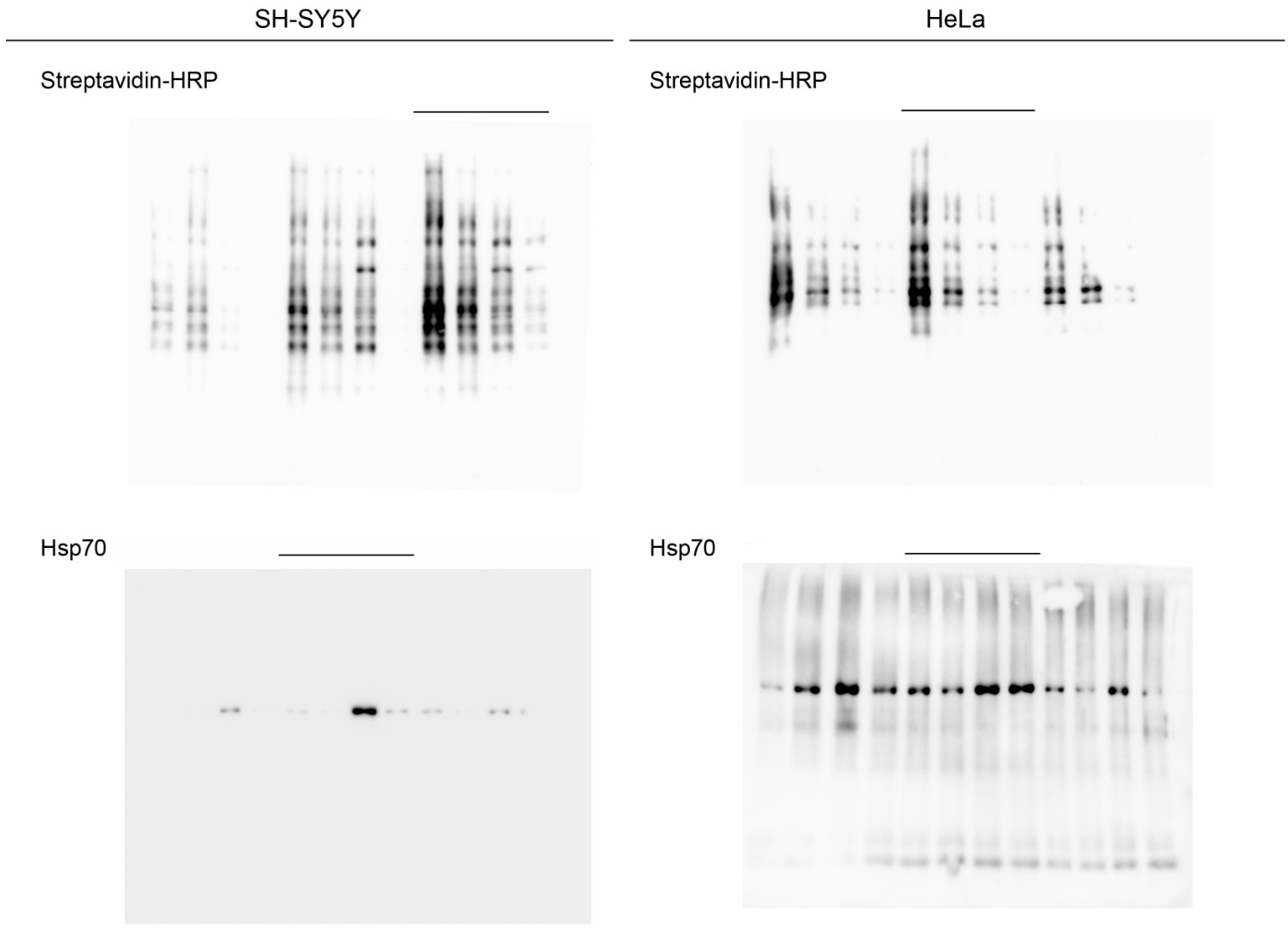

Figure S24. Original Western blots related to Figure 6 c, d. Vertical lines above the blot indicate the lanes included in the figure.
